# Energy-Period Profiles of Brain Networks in Group fMRI Resting-State Data: A Comparison of Empirical Mode Decomposition With the Short-Time Fourier Transform and the Discrete Wavelet Transform

**DOI:** 10.3389/fnins.2021.663403

**Published:** 2021-05-21

**Authors:** Dietmar Cordes, Muhammad F. Kaleem, Zhengshi Yang, Xiaowei Zhuang, Tim Curran, Karthik R. Sreenivasan, Virendra R. Mishra, Rajesh Nandy, Ryan R. Walsh

**Affiliations:** ^1^Cleveland Clinic Lou Ruvo Center for Brain Health, Las Vegas, NV, United States; ^2^University of Colorado, Boulder, CO, United States; ^3^University of Management & Technology, Lahore, Pakistan; ^4^School of Public Health, University of North Texas, Fort Worth, TX, United States; ^5^Muhammad Ali Parkinson Center at Barrow Neurological Institute, Phoenix, AZ, United States

**Keywords:** resting-state fMRI, empirical mode decomposition, EMD, intrinsic mode function, IMF, group ICA, functional connectivity, energy-period

## Abstract

Traditionally, functional networks in resting-state data were investigated with linear Fourier and wavelet-related methods to characterize their frequency content by relying on pre-specified frequency bands. In this study, Empirical Mode Decomposition (EMD), an adaptive time-frequency method, is used to investigate the naturally occurring frequency bands of resting-state data obtained by Group Independent Component Analysis. Specifically, energy-period profiles of Intrinsic Mode Functions (IMFs) obtained by EMD are created and compared for different resting-state networks. These profiles have a characteristic distribution for many resting-state networks and are related to the frequency content of each network. A comparison with the linear Short-Time Fourier Transform (STFT) and the Maximal Overlap Discrete Wavelet Transform (MODWT) shows that EMD provides a more frequency-adaptive representation of different types of resting-state networks. Clustering of resting-state networks based on the energy-period profiles leads to clusters of resting-state networks that have a monotone relationship with frequency and energy. This relationship is strongest with EMD, intermediate with MODWT, and weakest with STFT. The identification of these relationships suggests that EMD has significant advantages in characterizing brain networks compared to STFT and MODWT. In a clinical application to early Parkinson’s disease (PD) vs. normal controls (NC), energy and period content were studied for several common resting-state networks. Compared to STFT and MODWT, EMD showed the largest differences in energy and period between PD and NC subjects. Using a support vector machine, EMD achieved the highest prediction accuracy in classifying NC and PD subjects among STFT, MODWT, and EMD.

## Introduction

Functionally related regions of the resting brain have been shown to have a high degree of temporal correlation in blood-flow fluctuations as measured by blood-oxygenation level-dependent (BOLD) fMRI signal (Biswal et al., 1995). Using either seed-based or data-driven methods such as independent component analysis (ICA) or clustering methods, entire networks that fluctuate in synchrony have been found to constitute reliable and reproducible functional networks in the human resting brain (Beckmann, 2012; Lowe, 2012; Calhoun and de Lacy, 2017). These synchronous fluctuations may represent changes in local capillary blood flow secondary to fluctuations in neuronal firing rates within large distributed neural networks. From electrophysiological studies it is known that neural firing patterns occur over a wide range of frequency bands in the mammalian brain (Buzsáki and Draguhn, 2004). However, compared to the fast-neural firing response, the corresponding hemodynamic response that is detected in fMRI acts as a low-pass filter, is rather slow, and reaches a maximum several seconds later.

Frequency-specific analysis of resting-state networks have been carried out using bandpass filtering where the frequency intervals were specified using information from electrophysiological data (Gohel and Biswal, 2015) or simply by dividing the possible frequency band into equal bands that were conveniently specified by the user (Wu et al., 2008; Chen and Glover, 2015). The time-frequency dynamics of resting-state networks have also been studied using the continuous wavelet transform and recurring patterns of connectivity determined for specific frequency values (Yaesoubi et al., 2015).

An alternative method for finding frequency characteristics in resting-state data is by *Empirical Mode Decomposition* (EMD) [Niazy et al. (2011); Song et al. (2014)]. EMD is an adaptive time-frequency analysis method for studying the naturally occurring frequency bands in time series (Huang et al., 1998; Huang and Shen, 2014). EMD is particularly useful for non-stationary signals (such as fMRI data) and decomposes time series into nearly orthogonal modes spanning narrow, but partially overlapping, frequency bands. The oscillatory modes are called *intrinsic mode functions* (IMFs) and are obtained by a *sifting* algorithm. A sifting algorithm defines a process of how to separate larger features of a signal from smaller features in analogy to a mechanical sift. For EMD, the sifting starts by connecting the local maxima and minima of a signal through cubic splines to form the so-called upper and lower envelopes. Then, the average of the two envelopes is subtracted from the original signal, and finally, IMFs are obtained after applying this sifting process repeatedly. Further details of the sifting process are provided in Supplementary Appendix A. Instantaneous frequency-energy information for EMD can be obtained by applying the Hilbert Transform to IMFs. Unlike Fourier or wavelet based frequency analysis methods, neither predefined basis functions, stationarity, nor linearity are assumed in EMD, which makes it an ideal candidate for frequency-energy analysis in fMRI.

The purpose of this study is to investigate the frequency and energy characteristics of the time courses belonging to resting-state brain networks in fMRI data using EMD as an adaptive frequency-decomposition method and to compare results with conventional non-adaptive methods. Resting-state fMRI data are contaminated by motion, physiological noise, and other artifacts. These artifacts are difficult to handle and common artifact correction methods that use linear regression methods may be insufficient in providing artifact-free data without affecting the BOLD signal. In fact, in many research studies claiming high-frequency neural processes in resting-state data, artificial high frequencies may have been introduced by linear nuisance regression (Chen et al., 2017). Thus, our imaging data were not regressed against motion parameters, global signal or white matter signal. ICA has been shown to be a robust method when applied to group fMRI resting-state data and is less sensitive to subject motion. In general, spatial ICA can provide brain networks and associated time courses (so-called weights) which are less likely to contain artifacts, because neural-related signals are separated from different sources of noise (Beckmann, 2012). With the assumption that temporal profiles of spatial ICA reflect the underlying BOLD-fMRI time courses (McKeown et al., 1998), we investigated time courses associated with standard group spatial ICA brain networks and compared a decomposition of these time courses using the Short-Time Fourier Transform (STFT), the Maximal Overlap Discrete Wavelet Transform (MODWT), and EMD to determine if EMD has any advantages in characterizing brain networks (compared to STFT and MODWT). We like to emphasize that our EMD analysis in not limited to time series derived by ICA. Any resting-state spatial brain template (obtained by any means) can be used in a spatial regression application on the raw data to obtain corresponding time series of the network.

We would like to point out that EMD is not a method that is intended to replace spatial ICA or spatial clustering, which are used to obtain components representing brain networks fluctuating in synchrony. EMD should rather be considered as a *secondary analysis* to determine the frequency content of a signal of interest. In this study, we looked at time courses derived by group ICA and were interested to analyze these time courses further to determine the frequency content in specific frequency bands.

This is the first study where energy-period characteristics of fMRI resting-state networks are compared in relation to Gaussian noise data using three techniques, namely EMD, the *Short-Time Fourier Transform* (STFT), and the *Maximal Overlap Discrete Wavelet Transform* (MODWT). We chose the STFT (in dyadic frequency bands) and MODWT for comparison with EMD because 1) all three methods represent a form of a dyadic filterbank and preserve energy relationships, 2) the STFT is non-adaptive and based on fixed Fourier basis functions (which can be considered as a special but restrictive case of EMD); the MODWT allows more adaptivity but still is model-based by wavelet basis functions; and EMD is model-free, adaptive, and entirely data-driven. The advantages the EMD method offers for fMRI data analysis can be demonstrated through comparison to STFT and MODWT.

Furthermore, for each resting-state network, the resulting IMFs from above EMD analysis have very different characteristics in terms of their energy and period content. To identify similar IMFs over all subjects and resting-state networks, we carried out a clustering analysis and were able to group the resting-state networks based on their IMF energy and IMF period content into five different clusters leading to a data-driven characterization of all resting-state networks.

In a clinical application, we investigated whether STFT, MODWT, and EMD can find significant differences in temporal characteristics in a cohort of never-medicated early Parkinson’s Disease (PD) patients compared to normal controls (NC). Our benchmark for finding the optimal method is its ability to compute the largest difference in energy and period information between normal control and PD subjects. We chose the PD cohort because studies of the temporal characteristics of fMRI resting-state brain networks have shown abnormal spontaneous low-frequency content in PD (Hu et al., 2015) and abnormal whole-brain temporal network dynamics which correlate with PD symptoms (Zhuang et al., 2018), while implanted electrode studies have shown changes in synchronizations within and between brain regions as well as changes in phase-amplitude coupling between brain regions which correlate with clinical symptoms in PD (Yanagisawa et al., 2012; de Hemptinne et al., 2015). In our recently reported EMD study (Cordes et al., 2018), we also found that in many resting-state networks significant differences in temporal characteristics exist between PD and NC. We reanalyzed the data from this study using STFT and MODWT and provide a quantitative comparison to EMD by using a support-vector machine-learning approach to classify PD and NC.

## Theory

In order to explain the benefits of the EMD method, in particular how IMFs are computed by a sifting algorithm and how temporal characteristics (instantaneous energy and period (inverse frequency)) can be computed from these IMFs by the Hilbert Transform [for more information see Huang and Shen (2014)], we will briefly summarize EMD and contextualize this method within a broader theoretical framework. Given that many of these relationships are less well-known in the neuroscience community, we are providing reasonable detail, summaries, and explanations of these algorithms so that neuroscience researchers can use our findings to compute IMFs as well as energy and period characteristics. To provide a comparison to purely noise data, we show that EMD and white noise have a very unique relationship to energy and period content. In particular, *log*(energy) vs. log(period) are distributed linearly for white Gaussian noise. Using these noise characteristics, we can directly compare how fMRI signals of different resting-state networks relate to white noise characteristics. We also investigate the performance of EMD on simulated autoregressive first order (AR(1)) noise and compute the sensitivity of *log*(energy) and *log*(period) to changes in the AR(1) coefficient for STFT, MODWT and EMD. We explicitly show that *log*(period) depends strongly on the AR(1) coefficient when EMD is used. Furthermore, we also provide a more complicated simulation involving nonstationary data consisting of a superposition of three sine waves where amplitude and frequency changes in a nonlinear way with time, and we show how EMD separates these simulated data into three IMFs by ranking the instantaneous frequencies at each time point. Finally, we highlight that EMD is an approximate type of a dyadic filterbank decomposition where the decomposition is adaptive depending on the data and introduce STFT and MODWT as comparable but non-adaptive methods of dyadic filterbank decompositions.

### Empirical Mode Decomposition (EMD) and Intrinsic Mode Functions (IMFs)

EMD is a method that is defined by an algorithm to decompose a time series, whether nonstationary or nonlinear, into a set of IMFs. This decomposition is based on local characteristics of the time series. The Hilbert Transform is applied to the IMFs to compute instantaneous amplitude and frequency. Whereas Fourier and wavelet transforms use preassigned basis functions, the EMD basis functions are the *data-derived* IMFs. The EMD method operates at the scale of one oscillation and is adaptive to the local frequency content. An IMF represents a simple oscillatory mode but is more general than a harmonic function of *one* frequency component. In fact, an IMF can have variable amplitude and frequency content (in a narrow frequency band depending on the IMF index) along the time axis.

In general, an IMF is a function that must satisfy two conditions: (1) For the entire time series, an extremum must be followed by a zero crossing. (2) The mean value of the upper envelope (defined by the local maxima) and the lower envelope (defined by the local minima) is zero at every time point. A signal *x*(*t*) can be decomposed in terms of its *K* IMFs *f*_*k*_(*t*) by

(1) $$x\,\left(t\right)=\sum_{k=1}^{K}f_{k}\,\left(t\right)+r_{K}\,\left(t\right)$$

where *K* is the number of IMFs, *f*_*k*_(*t*) is the *k*-th IMF and *r*_*K*_(*t*) is a small monotone residual (trend) function. Supplementary Appendix A lists the basic algorithm for obtaining the IMF decomposition of a signal (A1). Furthermore, useful relationships regarding how instantaneous frequency and amplitude of IMFs are computed, are shown (A2). An illustration of EMD applied to a non-stationary signal can be found in A3 and the corresponding figure (Figure A1) in the Supplementary Appendix.

### Energy vs. Period Relationship of Intrinsic Mode Functions (IMFs)

The time series in fMRI data are known to contain structured as well as white noise sources. Since the IMFs are basis functions that are derived from the data rather than functions that satisfy given analytic expressions, it is important from a statistical perspective to understand the IMFs of simulated noise data so that IMFs of noisy signals can be compared to IMFs of pure noise data. Comparison to simulated noise data provides a reference standard of results obtained by EMD and allows a statistical significance to be associated with IMFs. Of particular importance is the *mean energy* as a function of the mean inverse of the frequency (*mean period*) for each IMF (Wu and Huang, 2004): the mean energy per time point, *E_k*, of the *k*-th IMF, *f*_*k*_(*t*), is defined by the mean instantaneous squared amplitude of the IMF per time point. This definition leads to

(2) $$E_{k}=\frac{1}{N}\,\sum_{t=1}^{N}f_{k}\,{\left(t\right)}^{2}$$

where *N* is the number of data points and the total energy per time point of the original time series is normalized to 1 resulting in $\sum_{k}E_{k}=1$. The mean period, *T*_*k*_, is defined by the mean value for the inverse of the instantaneous frequency obtained from the Hilbert Transform (Eq. A2), i.e.,

(3) $$T_{k}=\frac{1}{N}\,\sum_{t=1}^{N}\frac{1}{\nu_{k}\,\left(t\right)}.$$

However, due to outliers in the estimation of the instantaneous frequency spectrum (especially for frequencies close to zero), Eq. 3 does not provide robust values of *T*_*k*_. Instead, we determine the density of ν_*k*_(*t*) using kernel density estimation (with a Gaussian kernel), compute the cumulative density function (*cdf*) of the frequency distribution, and then discard all frequencies for which *cdf*(ν_*k*_) < 0.001 and *cdf*(ν_*k*_) > 1−0.001. From the density *h*(ν_*k*_), we calculate a more robust estimate of *T*_*k*_ by

(4) $$T_{k}=\frac{\int \frac{1}{\nu_{k}}\,h\,\left(\nu_{k}\right)\,d\,\nu_{k}}{\int h\,\left(\nu_{k}\right)\,d\,\nu_{k}}$$

where the integration is over all non-discarded frequencies ν_*k*_. For white Gaussian noise it has been shown (Wu and Huang, 2004) that:

(5) $$\log\left(E_{k}\right)=0.12-0.934\,\log\left(T_{k}\right)\approx -\text{log}\,\left(T_{k}\right).$$

Thus, *y* = *log*⁡(*E*_*k*_) as a function of *x* = *log*⁡(*T*_*k*_) is distributed approximately along the diagonal line *y* = −*x* for all IMFs of white noise data. The relationship in Eq. 5 is valid for a unit sampling rate.

### Quasi-Dyadic Filter Properties of EMD

A dyadic filterbank is defined as a *ratio-of-2 frequency-band* decomposition of a signal such that the frequency range of the different bands contains the intervals [$\frac{\nu_{N\,Q}}{2},\nu_{N\,Q}]$ for band 1, $\left[\frac{\nu_{N\,Q}}{4},\frac{\nu_{N\,Q}}{2}\right]$ for band 2, …, etc, where ν_*NQ*_ is the maximum frequency (Nyquist frequency). The MODWT and other discrete wavelet transforms typically represent exact dyadic filterbanks. EMD can also be considered as a tool that is *equivalent* to a dyadic filterbank in the frequency domain (Flandrin et al., 2004; see also Wu and Huang (2010) for other filterbank properties in relation to sifting operations). The corresponding IMFs are *nearly* orthogonal and differ in frequency content by a factor of approximately 2, which has been shown for a variety of broad-band noise data. However, for more complicated data such as fMRI data, this factor may vary depending on the frequency content since EMD is an adaptive method. For dyadic filter decompositions, in general, the first decomposition (such as IMF_1_ in EMD) has the highest frequency content and constitutes the widest frequency band whereas the last decomposition (IMF_*K*_ in EMD) has the lowest frequency content and represent the smallest frequency band. Because of the quasi-dyadic filter property, frequencies that are similar and differ by less than a factor of about 2 cannot be separated into different IMFs for data with broad-band frequency content. To establish the advantage of EMD over other dyadic filterbank methods, especially for white noise and AR(1) noise in relation to fMRI data, it is instructive to compare the filter-band properties of EMD, MODWT and STFT, as we show later.

### Other Time-Frequency Methods to Obtain Energy-Period Relationships of fMRI Time Courses

To compare results obtained with EMD, we compute energy-period relationships with the STFT and the MODWT using the orthogonal and compact supported Daubechies *db6* wavelets, which are commonly used in time-frequency analysis. Energy-period characteristics computed with these EMD, STFT and MODWT methods are compared using both simulated white Gaussian noise and also AR(1) noise time series.

#### Short-Time Fourier Transform (STFT)

The STFT of a discrete signal *x*(*t*) is defined by

(6) $$X\,\left(t,\nu \right)=\sum_{\tau =-\infty }^{\infty }x\,\left(\tau \right)\,w\,\left(\tau -t\right)\,e^{-i\,2\,\pi \,\nu \,\tau },$$

where *t*,τ are discrete time points, ν a frequency value, and *w*(*t*) represents a window function. The spectrogram of the STFT is given by |*X*(*t*,ν)|^2^ and can be conveniently computed in MATLAB^1^ by the spectrogram function for a given set of parameters (window length, overlap of windows, number of FFT points). The result is a time-frequency spectrum with energy of the frequencies as the third dimension at each time point. To obtain energy-period information that allows a comparison to EMD, we compute a dyadic decomposition of the spectrogram. In the *k*-th dyadic frequency band specified by the interval $\Delta \,\nu_{k}=\left[\frac{\nu_{N\,Q}}{2^{k}},\frac{\nu_{N\,Q}}{2^{k-1}}\right]$ with $\nu_{N\,Q}=\frac{1}{2\,T\,R}$ as the Nyquist frequency, we compute the instantaneous energy by

(7) $$E_{k}\,\left(t\right)=\frac{\sum_{\nu \in \Delta \,\nu_{k}}{\left|X\,\left(t,\nu \right)\right|}^{2}}{\sum_{\nu }{\left|X\,\left(t,\nu \right)\right|}^{2}}$$

and obtain the average energy by $E_{k}=\frac{1}{N}\,\sum_{t=1}^{N}E_{k}\,\left(t\right)$. Similarly, we define the instantaneous frequency ν_*k*_(*t*) in the *k*-th dyadic frequency band Δν_*k*_ by computing the energy-averaged frequency component according to

(8) $$\nu_{k}\,\left(t\right)=\frac{\sum_{\nu \in \Delta \,\nu_{k}}\nu \,{\left|X\,\left(t,\nu \right)\right|}^{2}}{\sum_{\nu }{\left|X\,\left(t,\nu \right)\right|}^{2}}$$

The average period *T*_*k*_can then be computed by Eq. 4 from Eq. 8. In this study we chose 64 time points for the window size, an overlap of 50% for adjacent windows, and 512 Fourier points in the spectrogram function. We empirically identified these parameters as reasonable for our purposes.

#### Maximal Overlap Discrete Wavelet Transform (MODWT)

The MODWT is an energy-preserving wavelet transform that produces coefficients related to variations of a time series over a set of dyadic frequency scales. Energy and period information are computed similarly as for EMD. Compared to the discrete wavelet transform, the MODWT is well defined at each time point and yields *K* detail functions *f*_*k*_(*t*) for *k* = 1,…,*K* at scales 2^1^ to 2^*K*^ and one approximation function *g*_*K*_(*t*) at scale 2^*K*^. Instantaneous frequency spectra can be computed by applying the Hilbert Transform to the MODWT detail coefficients for a given dyadic level (see for example Olhede and Walden, 2004), allowing a comparison with the EMD method for investigating average energy and period content for a dyadic filter decomposition of resting-state time courses. There are several different wavelets that can be used to obtain a dyadic frequency decomposition. We chose the popular Daubechies *db6* wavelet for the MODWT analysis. From the wavelet transform, the projections of the signals into different dyadic wavelet subspaces can be obtained by the Multi-Resolution Analysis function MODWTMRA in MATLAB. The original signal can be reconstructed by the sum of the signal projections using the MATLAB function MODWTMRA.

Wavelet coefficients or multiresolution analysis functions obtained by the MODWT are different from IMFs obtained by EMD. Contrary to IMFs, these functions cannot be locally represented by a single cosine function with time-varying frequency and amplitude characteristics where an extremum is always followed by a zero crossing, which leads to differences in the estimation of instantaneous frequency profiles for EMD and MODWT using the Hilbert Transform (see Figure A2 in the Supplementary Appendix). In the following, we use the term *Wavelet* synonymously with MODWT, for simplification.

## Materials and Methods

### Subjects

Subjects included 22 healthy undergraduate students (age 18–25, mean 23 years) with previous fMRI experience from the University of Colorado at Boulder. The local ethics committee approved the study protocol under IRB 13-0034, and all subjects provided written consent for the study. All subjects were right-handed. For fMRI, subjects were instructed to rest, keep eyes closed, and be as motionless as possible. Subject performance was monitored by a camera attached to the bore, and subjects were questioned after scanning regarding their wakefulness. Data where subjects fell into sleep were discarded and scanning was repeated on a different day.

### fMRI Acquisition

fMRI was performed in a 3.0 T Trio Tim Siemens MRI scanner equipped with a 32-channel head coil using the *Center for Magnetic Resonance Research (CMRR)* Simultaneous Multi Slice Multi Band (SMS MB) GE-EPI sequence with imaging parameters: MB 8, no parallel imaging, TR 765 ms, TE = 30 ms, flip 44 deg, partial Fourier 7/8 (phase), FOV = 19.1 × 14.2 cm, 80 slices in oblique axial orientation, resolution 1.65 mm × 1.65 mm × 2 mm, BW = 1724 Hz/pixel (echo spacing = 0.72 ms), 2380 time frames (30 min scanning duration). A single band reference image was also generated with the CMRR sequence. For distortion correction, two SE-EPI scans with same and opposite phase encodings were collected (same resolution, echo spacing and bandwidth as the GE-EPI). A 2D anatomical co-planar high resolution (0.4 mm × 0.4 mm × 1.5 mm) T2-weighted image and a 3D high resolution (1 mm × 1 mm × 1 mm) T1-weighted MPRAGE image were also collected.

### Data Preprocessing

Due to the short TR and multiband factor of 8, no slice-timing correction was performed to prevent *sinc* interpolation artifacts. All resting-state GE-EPI data and SE-EPI data were realigned to the single band EPI reference image in SPM12.^2^ Distortion correction was carried out with the *topup* routine in FSL^3^ to correct for the distortion in the phase encoding direction of the GE-EPI data. All fMRI data were normalized to the MNI-152 2 mm template using Advanced Normalization Tools (ANTS) software^4^ and spatially smoothed with an 8-mm Gaussian filter (which we found to be appropriate, given the increased white noise level of the data due to the multiband factor of 8, to prevent splitting and pixelated appearance of group ICA networks). The time series data of each subject were detrended by regression with discrete cosine wave functions (Frackowiak et al., 2004) to remove signal instabilities less than 0.01 Hz that were most likely introduced by heating of the preamplifier and the cooling cycle of the gradients (frequencies less than 0.01 Hz are of no known interest for fMRI resting-state networks). After detrending, the means of the time series data were removed and the obtained time series were variance normalized. Since motion estimation of the raw time series was small (rms motion <0.6 mm) for all subjects, we did not carry out an explicit motion regression to avoid frequency contamination of the data, since motion regression can induce high-frequency contamination (Chen et al., 2017).

### Frequency-Energy Characteristics of ICA Brain Networks

To carry out group ICA in a reasonable time with limited memory resources, data for each subject were reduced in the temporal domain to 200 components using Principal Component Analysis (PCA) and then stacked temporally. Then, a final PCA reduction on the stacked data to 30 components was done. Group spatial ICA (based on the FastICA algorithm with *tanh* nonlinearity (Hyvärinen, 1999)) was applied to obtain the major resting-state networks using customized code in MATLAB. We obtained 30 ICA components representing possible resting-state networks. Though the intrinsic dimensionality of fMRI resting-state data is unknown, our previous work (Cordes and Nandy, 2006) indicated that choosing a number much larger than 30 ICA components usually leads to splitting of traditional (low frequency) networks (like the DMN), whereas a number smaller than 30 does not provide a good representation of most common resting-state networks. Using spatial regression of the resting-state ICA group maps on the full (unreduced) group time series data, the group time series for all ICA components were obtained and associated with each subject after individual variance normalization (Beckmann et al., 2009; Filippini et al., 2009).

Once the spatial group ICA maps were obtained, the time series of the spatial ICA maps were decomposed into IMFs using EMD. For EMD we used the publicly available software package from P. Flandrin^5^ and limited the analysis to IMF components which covered the range of frequencies from 0.01 Hz to the Nyquist frequency (0.65 Hz). Since EMD is adaptive, the frequency partitioning of the time courses depends on the particular resting-state network. IMFs that cover frequencies less than 0.01 Hz are shown in some of the figures but were not used for any type of characterization of resting-state networks. In fact, IMFs with index number larger than 9 have mean frequencies within the very low drift range of fMRI signals (less than 0.01 Hz), have very low mean energies and are in general of no known importance in the characterization of low-frequency resting-state brain networks.

EMD is a *local* approach that is accurate over the period of 1 oscillation. As a consequence, IMFs at a time point t are determined using only information in a narrow interval (depending on the frequency content) of time point t. Thus, EMD can be applied on the *concatenated* group time series profiles rather than the individual subject-specific profiles with similar results when EMD is applied to the individual time series (with the exception of edge effects that occur at the beginning and end of each time series). After the IMFs are obtained, the corresponding IMF group spatial maps are calculated by temporal regression. The group spatial IMF maps resemble the spatial characteristics of each IMF. To obtain instantaneous frequency characteristics, the Hilbert Transform is applied to each IMF. The corresponding spectrum of each IMF is obtained with a standard kernel density estimation algorithm in MATLAB to study the frequency characteristics. Comparison of energy-period profiles of resting-state networks were then carried out using EMD, STFT, and MODWT methods.

### Clustering of Components Based on the Energy vs. Period Relationship

For EMD, we calculated for each IMF the average energy and period for each subject according to Eqs. 2, 4. Then, for each temporal profile of a resting-state ICA component, we defined a corresponding feature vector in IMF feature space that contains the average logarithmic energy and period information of all IMFs across all subjects according to

$$f_{p}=[\text{log}\left(E_{1\,p}^{\left(1\right)}\right),\ldots ,\log\left(E_{9\,p}^{\left(1\right)}\right),\ldots ,\text{log}(E_{1\,p}^{\left(22\right)},\ldots ,\log\left(E_{9\,p}^{\left(22\right)}\right),$$

$$\ldots ,\text{log}\left(T_{1\,p}^{\left(1\right)}\right),\ldots ,\log\left(T_{9\,p}^{\left(1\right)}\right),\ldots ,\text{log}\left(T_{1\,p}^{\left(22\right)}\right),\ldots ,\log\left(T_{9\,p}^{\left(22\right)}\right)],$$

where $E_{k\,p}^{\left(n\right)}$ and $T_{k\,p}^{\left(n\right)}$correspond to the average energy and average period of the *p*-th ICA component (network) of the *n*-th subject for IMF_*k*_, respectively. Since we have 22 subjects and 9 IMFs per subject, this feature vector has 22 × 9 × 2 = 396 entries for EMD. The feature matrix *X* contains the feature vectors of 30 ICA components, and thus has the dimension 30×396. For the other 2 methods (STFT, MODWT) we proceeded in a similar manner for forming the feature vectors. However, the number of components for STFT and MODWT is reduced to 7 because components larger than 7 are already in the drift range (T > 100 s). For all 30 ICA components, we combined the feature vectors into a similar feature matrix and carried out a *K-means* clustering using the squared Euclidean distance measure to obtain characteristic logarithmic energy-period profiles for each of the three analysis methods. The optimal number of clusters, *K*_*C*_, is determined by using a cross-validation approach with the leave-one-out method. In particular, *K-means* is run for a particular *K* on the feature matrix where one point *x*_*i*_ (one arbitrary row of the feature matrix) is deleted. Note that this *K* should not be confused with the previous notation for the number of IMFs. The minimum squared distance of the *i*-th left-out point (with *I* = total number of points) to the *K* centroids $c_{k}^{\left(i\right)}$ for *k* = 1*toK* is calculated, and the process is repeated using a different left-out point. The average of the minimum squared distances is then the cross-validation error *CVE*(*K*) given by

(10) $$C\,V\,E\,\left(K\right)=\frac{1}{I}\,\sum_{i=1}^{I}m\,i\,n_{k\in \left\{1,\ldots ,K\right\}}\,d\,{\left(x_{i},c_{k}^{\left(i\right)}\right)}^{2}$$

and can be plotted as a function of *K*. The first local minimum of *CVE*(*K*) as *K* is increased starting from 1 then provides a cross validation measure to find an optimal number of clusters *K*_*C*_. Alternatively, the point of maximum curvature of *CVE*(*K*) can be used as well to determine *K*_*C*_. For the Boulder data, both values coincided.

**FIGURE 1:**
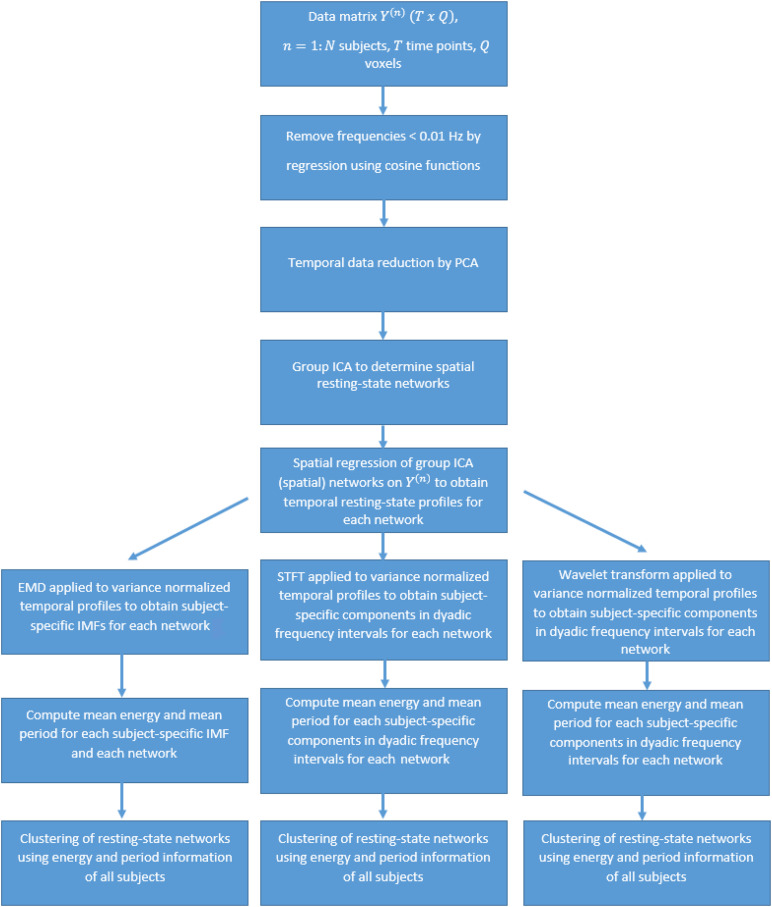
Flow chart of the data analysis.

To visualize the clustering results in a low-dimensional space, we use PCA applied to the feature matrix *X* (containing 30 ICA components x 396 energy/period features for EMD or 30 ICA components x 308 energy/period features for STFT/MODWT). We computed eigenvalues and eigenvectors by solving the eigenvalue equation

(11) $$X^{T}\,X\,V=V\,\text{Λ}$$

where the matrix *V* (size 396×30 for EMD, size 308×30 for STFT and MODWT) represents the 30 different eigenvectors in its columns and Λ is the associated diagonal eigenvalue matrix. The transformed feature matrix in PCA space then becomes

(12) $$\tilde{X}=X\,V$$

and has the dimension of *30* resting-state networks ×30 principal components. For a low-dimensional graphical representation of the feature matrix using PCA, we provide figures using only the first two PCA components (with the largest eigenvalues). Figure 1 shows a flow chart of the entire data analysis.

### Clinical Application to Early-Stage Never-Medicated PD (PPMI Data)

We reanalyzed clinical data from our recently published EMD study (Cordes et al., 2018) by using the STFT and MODWT method as a comparison to EMD. These data were obtained from the publicly available anonymized Parkinson’s Progression Markers Initiative (PPMI) database (Marek et al., 2011). We included 18 NCs (14 Male (M); age: 64.25 ± 9.78 years (mean ± SD), years of education 16.72 ± 2.67 years) and 18 newly diagnosed, early-stage, and never medicated PD subjects (10 M; age: 57.11 ± 11.63 years; years of education: 17.00 ± 2.77 years; disease duration: 0.83 ± 0.84 years) in our analysis. Differences in gender, age, and years of education were not statistically significant. Briefly, all subjects underwent resting-state fMRI scans on 3T Siemens scanners (8 min 24 s, EPI, 210 time points, TR = 2,400 ms, TE = 25 ms, FOV = 22.4 cm, flip angle = 80deg, resolution = 3.3 mm^3^ × 3.3 mm^3^ × 3.3 mm^3^, 40 axial slice). The first 5 time points (12 s) were removed to allow the MR signal to achieve T1 equilibrium. EPI data were slice-timing corrected and realigned to the mean echo-planar image in SPM12^6^, further co-registered to the subject T1 space, and then normalized to the standard MNI-152 2 mm-template using ANTs software. fMRI data were further spatially smoothed using an 8 mm 3D-Gaussian filter and drift frequencies less than 0.01 Hz were removed. Group ICA was performed by stacking all data (NC+PD) in the temporal domain to obtain 30 resting-state networks. Then, similar to the resting-state analysis, spatial regression was used on the networks of the group time series data to obtain the time series for the NC group and for the PD group. STFT, MODWT, and EMD were used to decompose the time series of the resting-state networks into components covering a frequency range from 0.01 Hz to the Nyquist frequency (0.5/TR) of the data. For each component and method, the average instantaneous energy, period, and their standard deviations were computed for NC and PD.

To quantify the significance of the energy-period relationships, we carried out a *leave-one-out* classification with a support vector machine (SVM). The input to the SVM contained log(energy) and log(period) feature vectors for decompositions 1 to 5. One subject was left out in the training of the SVM and the prediction accuracy (*PA*), defined by

P⁢A=p⁢(c⁢o⁢r⁢r⁢e⁢c⁢t⁢c⁢l⁢a⁢s⁢s⁢i⁢f⁢i⁢c⁢a⁢t⁢i⁢o⁢n)

(13) =p⁢(N⁢C)⁢p⁢(N⁢C|N⁢C)+p⁢(P⁢D)⁢p⁢(P⁢D|P⁢D),

was computed on the *left-out* group member. In this equation, *p*(*NC*) = 0.5 and *p*(*PD*) = 0.5 are the prior probabilities since the groups were balanced. The conditional probability *p*(*NC*|*NC*): = *p*(*classifiedasNC*|*truelabelisNC*)refers to the scenario that the group member was labeled NC and the classification resulted in the label NC. A similar definition holds for *p*(*PD*|*PD*): = *p*(*classifiedasPD*|*truelabelisPD*). The approach was repeated for all possible *leave-one-out* combinations (i.e., 36 times) to arrive at an average prediction accuracy. The null distribution for the leave-one-out method is obtained by a random permutation of the class labels and follows a binomial distribution with probability density

(14) $$p\,\left(k\right)=\left(\begin{array}{c} 36\\ k \end{array}\right)\,{\left(\frac{1}{2}\right)}^{k}\,{\left(\frac{1}{2}\right)}^{36-k}$$

where *k* ∈ {0,1,2,…,36} is the number of successes to obtain the classification of *PD* in 36 trials. The corresponding prediction accuracy at level 1-α is $P\,A=\frac{\hat{K}}{36}$ where $\hat{K}=a\,r\,g\,\min_{K}\sum_{k=0}^{K}p\,\left(k\right)\geq 1-\alpha .$

## Results

### Energy-Period Relationship of White Gaussian Noise and AR(1) Noise: Comparing STFT, MODWT and EMD

Noise in short TR fMRI data is dominated by white Gaussian noise and strong autoregressive noise (Chen et al., 2019). We simulated 1000 Gaussian white noise time series data ε(*t*) with the same high-resolution TR (765 ms) and number of data points as the fMRI data to represent a simple noise process and used EMD to obtain the first 9 IMFs. Average energy and period were calculated according to Eqs. 7, 9. We repeated the simulation for different autoregressive Gaussian noise processes of order 1 (AR(1)) to show the effect of strong first-order temporal correlations on the energy-period relationships of IMFs. In particular, we created time series data according to the model

**FIGURE 2:**
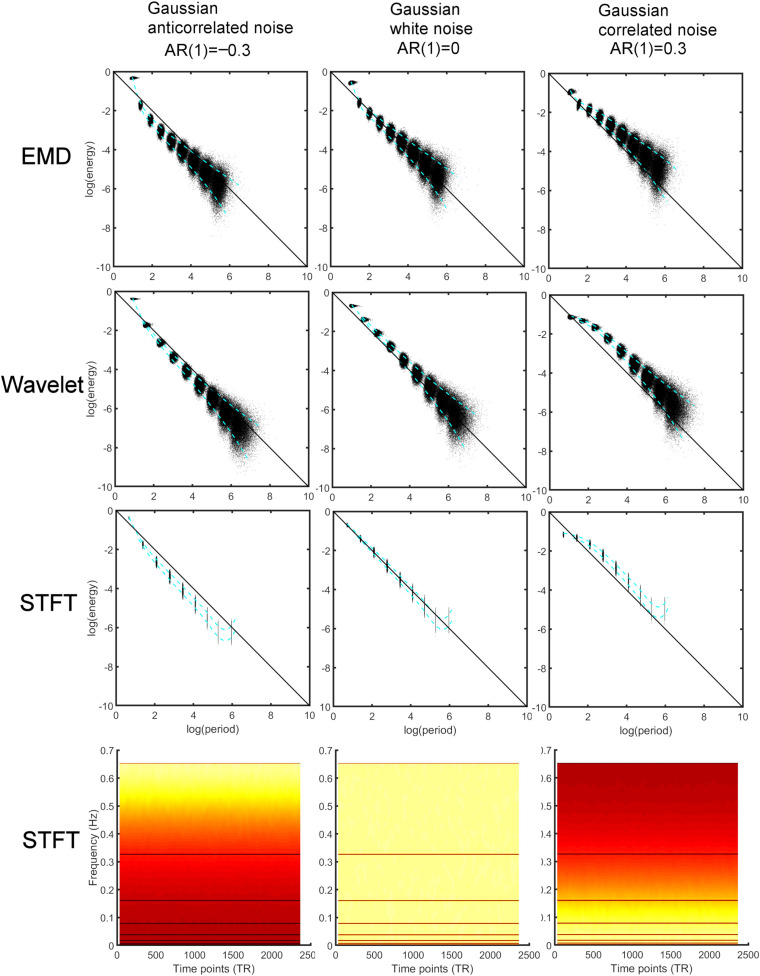
Comparison of energy-period relationship of different noise processes [Gaussian anticorrelated AR(1) noise **(left)**, Gaussian white noise **(middle)**, Gaussian correlated AR(1) noise **(right)**] for 1000 simulated time series data (TR 0.765 s, 2367 time points). Energy-period information was obtained using different analysis methods. *Top*: Empirical Mode Decomposition (EMD) determines Intrinsic Mode Functions (IMFs) of which the first 9 IMFs were used to obtain energy-period information. The blue dotted lines represent the 5 and 95% of the energy-period distribution for all IMFs. *Second from top*: The Maximal Overlap Discrete Wavelet Transform (MODWT) leads to similar energy-period profiles for the first nine detail functions. *Third from top*: Energy-period relationship in dyadic frequency bands determined by the Short-Time Fourier Transform (STFT). *Bottom*: Spectrogram using the STFT. The horizontal lines indicate the different dyadic frequency bands. Hot (yellow) color indicates larger intensity (energy). The larger frequency bands have larger energy content. Note that a negative AR(1) process leads to high-frequency noise whereas a positive AR(1) leads to low-frequency noise indicated by the yellow color.

**FIGURE 3:**
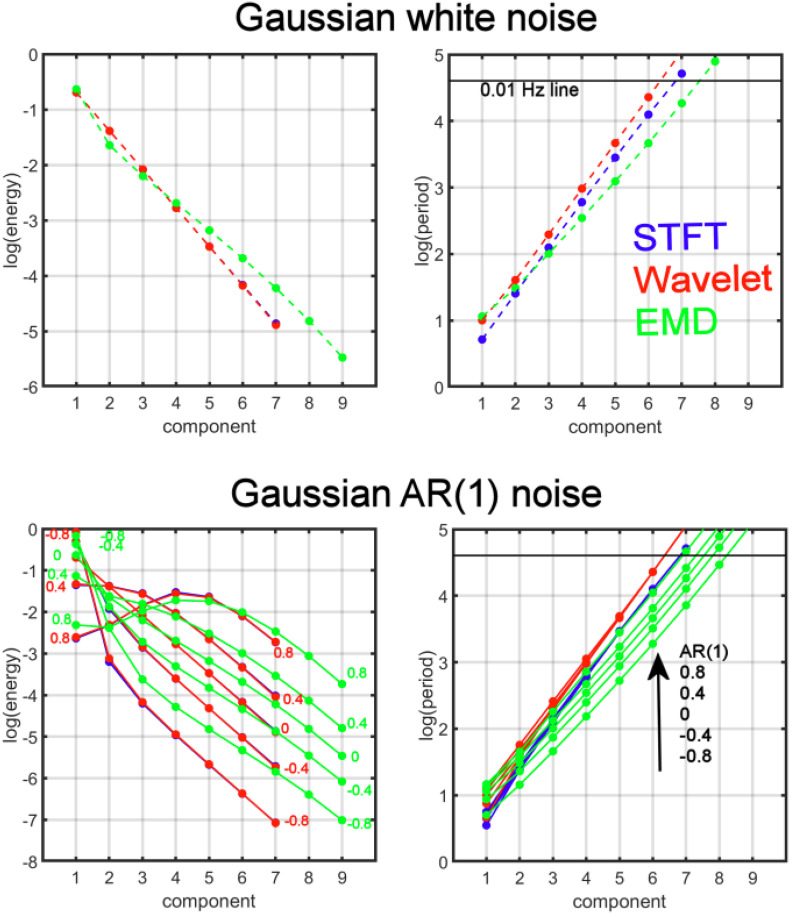
Comparison of the average energy and period distributions for simulated Gaussian white noise **(top)** and AR(1) noise **(bottom)** for AR(1) values ϕ = {−0.8,−0.4, 0, 0.4, 0.8} as a function of the decomposition level (component) and for three different analysis methods: Empirical Mode Decomposition (EMD, green), Maximal Overlap Discrete Wavelet Transform (Wavelet, red), and Short-Time Fourier Transform (STFT, blue). For EMD the different components are the IMFs, whereas for the wavelet transform the detail levels are the components, and for STFT the different dyadic frequency intervals are the components. The numbers in the figure indicate the values of the AR(1) coefficient. The black horizontal line corresponds to the 0.01 Hz line indicating the border to the drift frequency range in fMRI. Components with periods above this line are most likely associated with artifacts and are of no interest in this study. Note that *log(period)* shows approximately linear dependence as a function of the components for all methods. In particular, the AR(1) coefficient associated with the lines for *log(period)* increases in the direction of the arrow (toward larger periods). The *log(period)* dependence on the AR(1) coefficient is largest for EMD (see large spread of the 5 green lines) and very small for STFT and wavelet, especially for components larger than 1 (with a spread close to zero for the 5 red lines (or 5 blue lines)). Also, the lines for EMD have a smaller slope when compared to STFT and MODWT. The *energies* for STFT and MODWT are the same (red and blue energy curves overlap).

(15) η⁢(t)=ϕ⁢η⁢(t-1)+ε⁢(t),

where we chose the autocorrelation strength ϕ ∈ [−0.8,−0.7,…, 0.8] to obtain different AR(1) noise. Figure 2 (top) shows the energy-period relationship for a small sample of the different noise processes (using ϕ = {0,−0.3, 0.3}). Each data point in the figure represents a feature point derived from the IMF time series. All energy-period data points belonging to a specific IMF form an oval-shaped cluster of points. With increasing AR(1) coefficient, the energy-period spectrum is shifted upward for IMFs 2–9, whereas for IMF_1_ the shift is downward toward the diagonal line. The blue dotted lines represent 5% and 95% of the energy-period distribution for all IMFs. We also compare these results with the MODWT according to Section “Maximal Overlap Discrete Wavelet Transform (MODWT)” for the first nine detail functions that corresponds to dyadic frequency bands. The periods for the detail functions are larger for the same detail index when compared to the IMF with the same index (Figure 2, second from top). Finally, we compute logarithmic energy-period relationships in dyadic frequency bands by using the STFT according to Eqs. 4, 7, and 8 (Figure 2, third from top) and also show the spectrogram using the STFT (Figure 2, bottom). The period for each frequency band is fixed and only the energy varies. To show the differences in the profiles for average logarithmic energy *E*_*k*_ and period *T*_*k*_in Figure 2, we provide in Figure 3 a comparison of the three methods for white noise data, as a function of the decomposition level *k* and a function of the AR(1) coefficient. After a 2-parameter (*A*,*B*) least square fit of the energy data using the linear parameterization

(16) $$l\,o\,g\,E_{k}=A+B\,k,$$

which is equivalent to *E*_*k*_ = *ab*^*k*^(for constants *A* = *loga*,*B* = *logb*), and similarly for the period *T*_*k*_ data, we determined *a* and *b* (from parameters *A*,*B*) and obtained approximately the following relationships for white Gaussian noise:

$$\left(\begin{array}{c} E_{k}\,\left(S\,T\,F\,T\right)\\ E_{k}\,\left(M\,O\,D\,W\,T\right)\\ E_{k}\,\left(E\,M\,D\right) \end{array}\right)=\left(\begin{array}{c} 0.50^{k}\\ 0.50^{k}\\ 0.71^{*}\,0.57^{k} \end{array}\right)\;\text{and}$$

(17a) $$\left(\begin{array}{c} T_{k}\,\left(S\,T\,F\,T\right)\\ T_{k}\,\left(M\,O\,D\,W\,T\right)\\ T\,\left(E\,M\,D\right) \end{array}\right)=\left(\begin{array}{c} 1.07^{*}\,1.96^{k}\\ 1.32^{*}\,1.98^{k}\\ 1.43^{*}\,1.76^{k} \end{array}\right)$$

This analysis shows that EMD deviates from a perfect dyadic decomposition of the time series (since for EMD the factor 1.76 is significantly different from 2, whereas for STFT and MODWT the factor is very close to the expected value of 2). Similarly, for white noise the energy content by EMD is reduced by a factor of 0.57 with each decomposition level, which is different than the expected value of 0.5 (for STFT and MODWT). A consequence of the reduced factor of 1.76 for EMD is that the drift frequency range for fMRI data (which is about 0.01 Hz) is obtained for a larger *k*. For example, the STFT and MODWT methods achieve a period larger than 100 s for *k*≥7 whereas for EMD the value is *k*≥8.

Next, we investigated the energy and period relationships for AR(1) noise. Since log(period) shows an approximate linear dependence on the components for all AR(1) noise scenarios, we expanded our model to determine the dependence of *log(period) on the AR(1*) coefficient ϕ. A suitable model is given by *logT*_*k*_ = *A* + *Bk* + *C*ϕ + *Dk*ϕ with constants *A*,*B*,*C*,*D*. We obtained the following relationships

$$\left(\begin{array}{c} \log\left(T_{k}\right)\,\left(S\,T\,F\,T\right)\\ \text{log}\,\left(T_{k}\right)\,\left(M\,O\,D\,W\,T\right)\\ \log\left(T_{k}\right)\,\left(E\,M\,D\right) \end{array}\right)$$

(17b) $$\,=\left(\begin{array}{c} 0.0680+0.6707\,k+0.1048\,\phi -0.0178\,k\,\phi \\ 0.2742+0.6811\,k+0.2233\,\phi -0.0400\,k\,\phi \\ 0.3235+0.5671\,k+0.2570\,\phi +0.0277\,k\,\phi \end{array}\right).$$

We define the log(period) noise sensitivity, $S_{k}^{T}$, to the AR(1) coefficient ϕ by calculating

(18) $$S_{k}^{T}=\left|\frac{\partial \text{log}\,\left(T_{k}\right)}{\partial \phi }\right|.$$

We found that on average (over all components) the noise sensitivity is approximately

(19a) $$\left(\begin{array}{c} S_{k}^{T}\,\left(S\,T\,F\,T\right)\\ S_{k}^{T}\,\left(M\,O\,D\,W\,T\right)\\ S_{k}^{T}\,\left(E\,M\,D\right) \end{array}\right)=\left(\begin{array}{c} 0.04\\ 0.09\\ 0.40 \end{array}\right).$$

Thus, EMD is more than 4 times as sensitive as MODWT and 10 times as sensitive as STFT to the strength of noise correlations (ϕ). Similar computations can also be carried out for log(energy) sensitivity $S_{k}^{E}$, however, the relationships are more complicated since the log(energy) dependence for AR(1) noise has significant nonlinear contributions (see Figure 3). We carried out a numerical differentiation to determine $S_{k}^{E}$ and obtained

(19b) $$\left(\begin{array}{c} S_{k}^{E}\,\left(S\,T\,F\,T\right)\\ S_{k}^{E}\,\left(M\,O\,D\,W\,T\right)\\ S_{k}^{E}\,\left(E\,M\,D\right) \end{array}\right)=\left(\begin{array}{c} 1.8\\ 1.8\\ 1.5 \end{array}\right),$$

which shows that log(energy) computed by EMD is by a factor of about 0.37 less sensitive to the AR(1) coefficient than STFT and MODWT.

### Energy-Period Relationship of a Typical Resting-State Network: The Default Mode Network (DMN)

**FIGURE 4:**
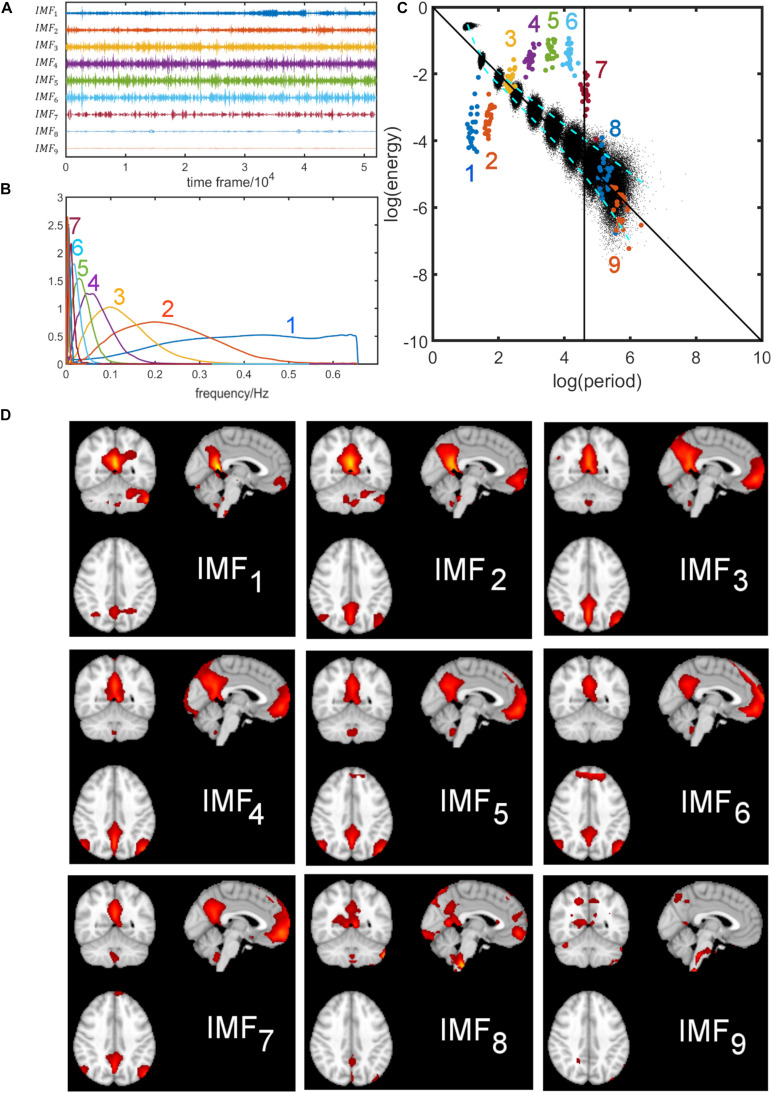
Decomposition of the default mode network (DMN) using empirical mode decomposition. The time courses for all subjects were concatenated. Top left figure **(A)** shows the first nine intrinsic mode functions (IMFs). The corresponding frequency distributions are shown in figure **(B)**. IMF_1_ has a wide high-frequency spectrum whereas the higher-order IMFs have a narrow-band low-frequency spectrum. The figure on the top right **(C)** shows the energy-period relationship of the IMFs of the DMN superimposed on the white noise spectrum (black dots). Each colored dot represents a subject. The black vertical line corresponds to the drift-frequency cut-off of 0.01 Hz. Information to the right of this line correspond to scanner-related artifacts and is of no interest in this study. In the bottom figure **(D)**, the spatial components associated with each IMF are shown. The DMN is seen more clearly in IMFs 2–7 than in IMFs 1,8,9.

Figure 4 shows the results obtained for the DMN using EMD applied to the concatenated network time courses of all Boulder subjects. The concatenated time series of the DMN was decomposed by EMD into nine IMFs, and the IMFs for each subject were extracted. We chose to use the concatenated time profile rather than conducting EMD for each individual subject because the concatenated approach appeared to be more stable for EMD by reducing the variance of parameters of interest (i.e., log(energy) and log(period)) slightly. Then, the corresponding frequency distribution was estimated using Eqs. A1–A3. The computed time series and frequency distribution are shown in Figures 4A,B, respectively. IMF_1_ has a wide high-frequency spectrum, whereas the higher-order IMFs have a narrow-band low-frequency spectrum. Average energy and period were calculated for each subject according to Eqs. 2, 4. Figure 4C shows the logarithmic energy vs. period relationship of the IMFs of the DMN superimposed on the white noise spectrum (black dots). IMFs with the same index have the same color and consist of 22 subject-specific points with narrow spread in period (Figure 4C). For each IMF, we also calculated the corresponding spatial map by regression using the concatenated IMF for all subjects (Figure 4D). The DMN is seen clearly in IMFs 2–7, whereas the DMN is incompletely obtained from IMFs 1,8,9. A listing of the peak frequency, FWHM, frequency range of the first nine IMFs, and the spatial similarity of each IMF to the group ICA map is provided in Supplementary Material 1 for the DMN.

### Comparison of Energy-Period Profiles for Five Common Networks That Differ in Their Frequency Content

**FIGURE 5:**
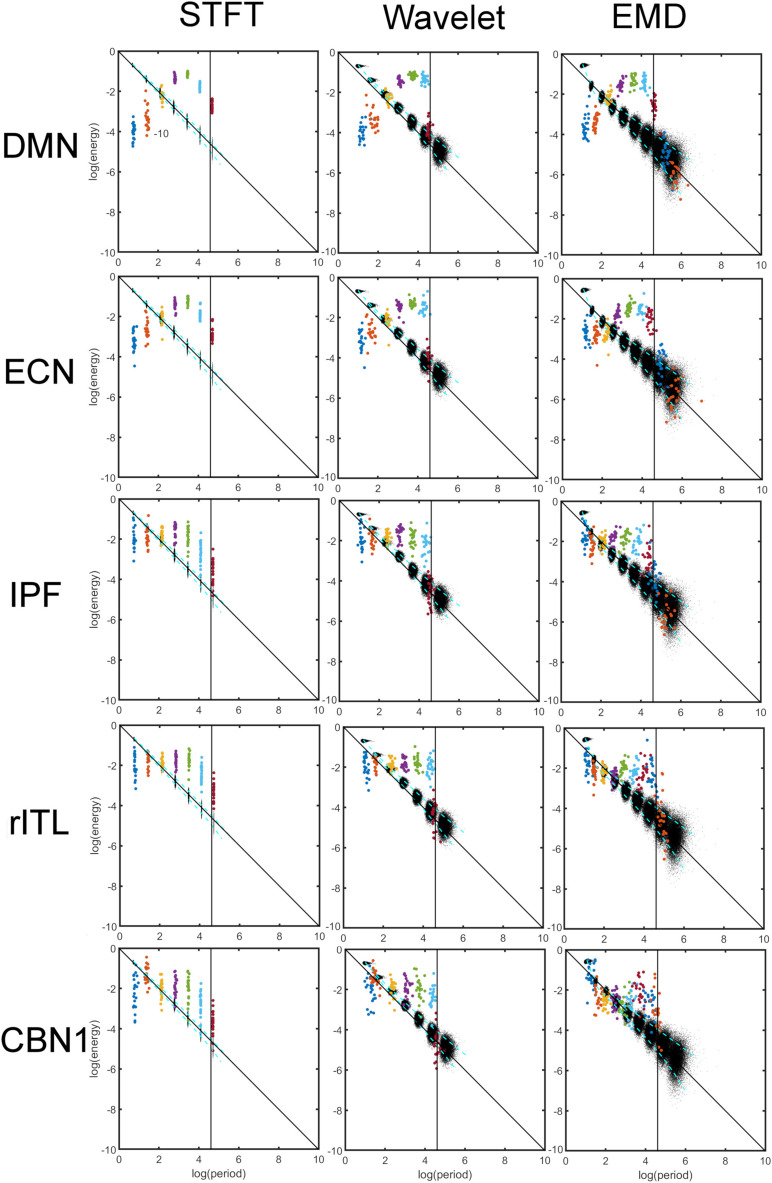
Comparison of energy and period profiles using three different analysis methods (Short-Time Fourier Transform (STFT), Maximal Overlap Discrete Wavelet Transform (MODWT), Empirical Mode Decomposition (EMD)) applied to five different resting-state networks (Default Mode Network (DMN), Executive Control Network (ECN), Inferior Prefrontal Network (IPF), right Inferior Temporal Network, Cerebellar Network 1 (CBN1). As before (see Figures 2, 4), the black dots represent a data decomposition of white Gaussian noise. Dots of the same color correspond to the same component of the decomposition, i.e., dyadic frequency interval decomposition for STFT, detail level decomposition of the wavelet transform, and intrinsic mode function (IMF) decomposition for EMD. There are 22 dots with the same color corresponding to the 22 subjects studied. The vertical line corresponds to the cut-off frequency of 0.01 Hz, and information to the right of this line (i.e., frequencies lower than 0.01 Hz) is of no interest in this study. Note that these 5 networks were chosen to be representative networks of the clusters 1 to 5 in Figure 10.

Figure 5 shows a comparison of energy and period profiles using the STFT, MODWT and EMD applied to the DMN, Executive Control Network (ECN), Inferior Prefrontal Network (IPF), right Inferior Temporal Network, and Cerebellar Network 1 (CBN1). These networks were chosen based on their different frequency content. For example, the DMN is mostly characterized by low frequencies (large periods) whereas the CBN1 has a large contribution of high frequencies (low periods). The other chosen networks are in between these extreme characteristics. Since the frequency content is different for different networks and EMD is an adaptive method, the number of decompositions (components) obtained by EMD (but not for STFT and MODWT) is in general different for different networks. Thus, the STFT and MODWT methods give six important components for all five networks with frequency range larger than 0.01 Hz, whereas the number of components (IMFs) for EMD is different for the five networks. In particular DMN has six, ECN and IPF have seven, rITL has eight, and CBN1 has nine IMFs.

**FIGURE 6:**
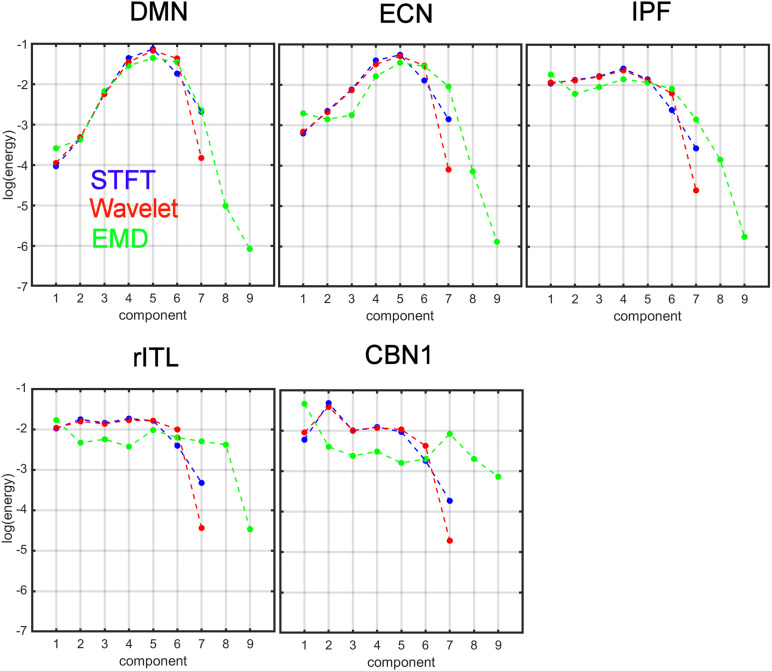
Comparison of energy profiles averaged over all subjects using Short-Time Fourier Transform (STFT), Maximal Overlap Discrete Wavelet Transform (MODWT), and Empirical Mode Decomposition (EMD) for the different resting-state networks from Figure 5. Note that these 5 networks were chosen to be representative networks of the clusters 1 to 5 in Figure 10.

Figure 6 compares the energy profiles, averaged over all subjects (computed from Figure 5). The DMN and ECN show a bell-shaped curve for the different decomposition levels, whereas for IPF, rITL and CBN1 the energy is more flat for the first few decompositions 1 to 5. For these five networks IMF_1_ (component 1 in green curve in Figure 6) has the highest energy compared to the first component of STFT and MODWT (for example the DMN has *log*(*E*) = −3.60*forEMD*, −3.95*forMODWT*,−4.00*forSTFT*).

**FIGURE 7:**
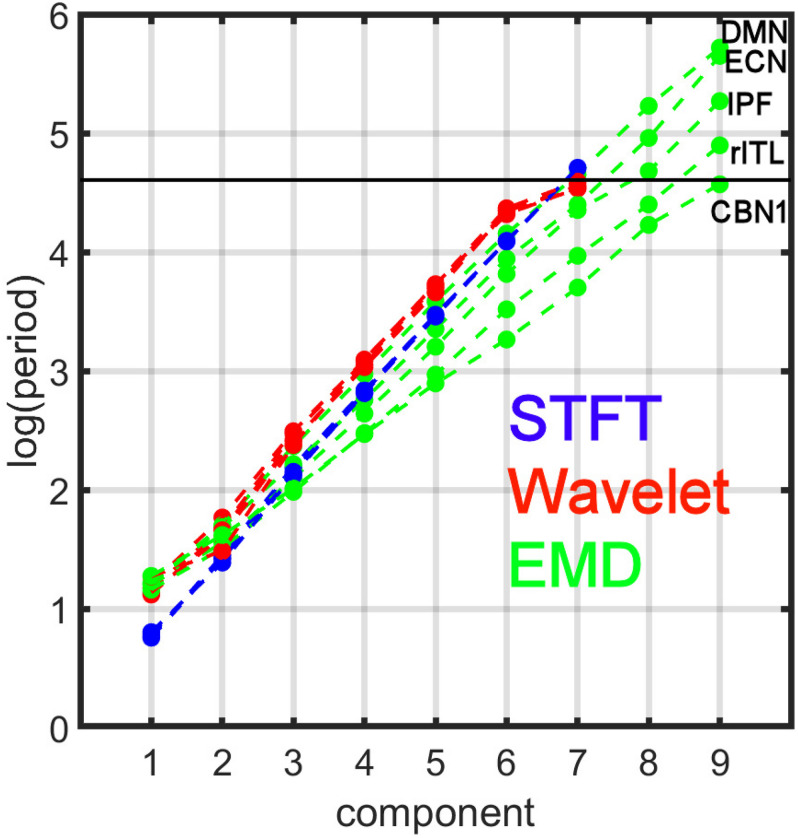
Comparison of period profiles averaged over all subjects using Short-Time Fourier Transform (STFT), Maximal Overlap Discrete Wavelet Transform (MODWT), and Empirical Mode Decomposition (EMD) for the different resting-state networks from Figure 5. Different resting-state networks show deviations to a perfect dyadic decomposition for EMD. Low-frequency networks (for example DMN) show the least deviation of EMD with STFT and MODWT, whereas high-frequency networks (for example CBN1) show largest deviations (similar to Figure 3). Note that these 5 networks were chosen to be representative networks of the clusters 1 to 5 in Figure 10. See also corresponding Tables 1, 2.

Figure 7 compares the period profiles, averaged over all subjects (computed from Figure 5). Using EMD, the profiles for the networks of DMN, ECN, IPF, rITL, and CBN1 have very different slopes for the different decomposition levels, showing that EMD is a *network-specific* frequency-adaptive decomposition method contrary to STFT and MODWT. Specifically, the period of the IMFs decreases from DMN, ECN, IPF, rITL, to CBN1 (in this order). The corresponding slopes *m* of the line *log*⁡(*T*_*k*_)=*const*.+*mk* are approximately 1.8 (DMN), 1.75 (ECN), 1.7 (IPF), 1.6 (rITL) and 1.5 (CBN1), showing that the *EMD decomposition becomes less dyadic for networks with high frequency contents*. Table 1 provides the goodness of fit information for *R*^2^, which is close to 1 for all networks, justifying our parameterization of *log*⁡(*T*_*k*_). We also investigated whether the lines for the five networks are significantly different from each other. Table 2 shows the statistics (p value and effect size) for comparing DMN vs. ECN, ECN vs. IPF, IPF vs. rITL, and rITL vs. CBN1 over all subjects. Only EMD gave a very significant difference (p < 0.001) with a medium or large effect size for these comparisons.

**TABLE 1:** *R*^2^ goodness of the linear fit for *log*⁡*T*_*k*_ for the different resting-state networks in Figures 5, 7.

	**STFT**	**Wavelet**	**EMD**
DMN	0.998	0.981	0.993
ECN	0.999	0.982	0.988
IPF	0.999	0.985	0.987
rITL	0.999	0.982	0.990
CBN1	0.998	0.973	0.981

*A linear model was used for fitting the period *log*⁡*T*_*k*_ to *k* according to *log*⁡*T*_*k*_ = *kA* + *B* + ε, where *A* and *B* are regression coefficients and ε is an error term.*

**TABLE 2:** Cohen’s effect size and p value for pairwise comparison of *log*⁡*T*_*k*_ for the different resting-state networks in Figures 5, 7.

	**STFT**	**Wavelet**	**EMD**
DMN vs. ECN	*f*^2^= 0.0026	*f*^2^= 0.0421	*f*^2^= 0.0924 (*)
	*p* = 0.53	*p* = 0.01	*p* = 3×10^−5^ (*)
ECN vs. IPF	*f*^2^= 0.0492	*f*^2^= 0.0187	*f*^2^= 0.1525 (*)
	*p* = 0.01	*p* = 0.09	*p* = 1×10^−7^ (*)
IPF vs. rITL	*f*^2^= 0.0446	*f*^2^= 0.0063	*f*^2^= 0.5315 (*)
	*p* = 0.01	*p* = 0.33	*p* = 6×10^−20^ (*)
rITL vs. CBN1	*f*^2^= 0.0203	*f*^2^= 0.0008	*f*^2^= 0.7084 (*)
	*p* = 0.08	*p* = 0.72	*p* = 1×10^−24^ (*)

*The significance that adjacent networks in Figure 7 are different was computed by an F test of a linear difference fit using *log*⁡*T*_*k*_(*network* 1)−*log*⁡*T*_*k*_(*network* 2) = *kA* + *B* + ε over all subjects, where *A* and *B* are regression coefficients and ε is an error term. Only EMD shows a very significant difference (*p* < 0.001) with a medium or large effect size (indicated by a star). In general, *f*^2^≈0.1^2^ = 0.01 is considered a small effect size, *f*^2^≈0.25^2^ = 0.0625 a medium effect size, and *f*^2^≈0.4^2^ = 0.16 a large effect size (en.wikipedia.org/wiki/Effect_size#cite_note-CohenJ1988Statistical-8). The 5 networks were chosen to be representative networks of the clusters 1 to 5 in Figure 10, i.e., DMN (cluster 1), ECN (cluster 2), IPF (cluster 3), rITL (cluster 4), CBN1 (cluster 5).*

### Clustering of Energy-Period Profiles of All ICA Components

**FIGURE 8:**
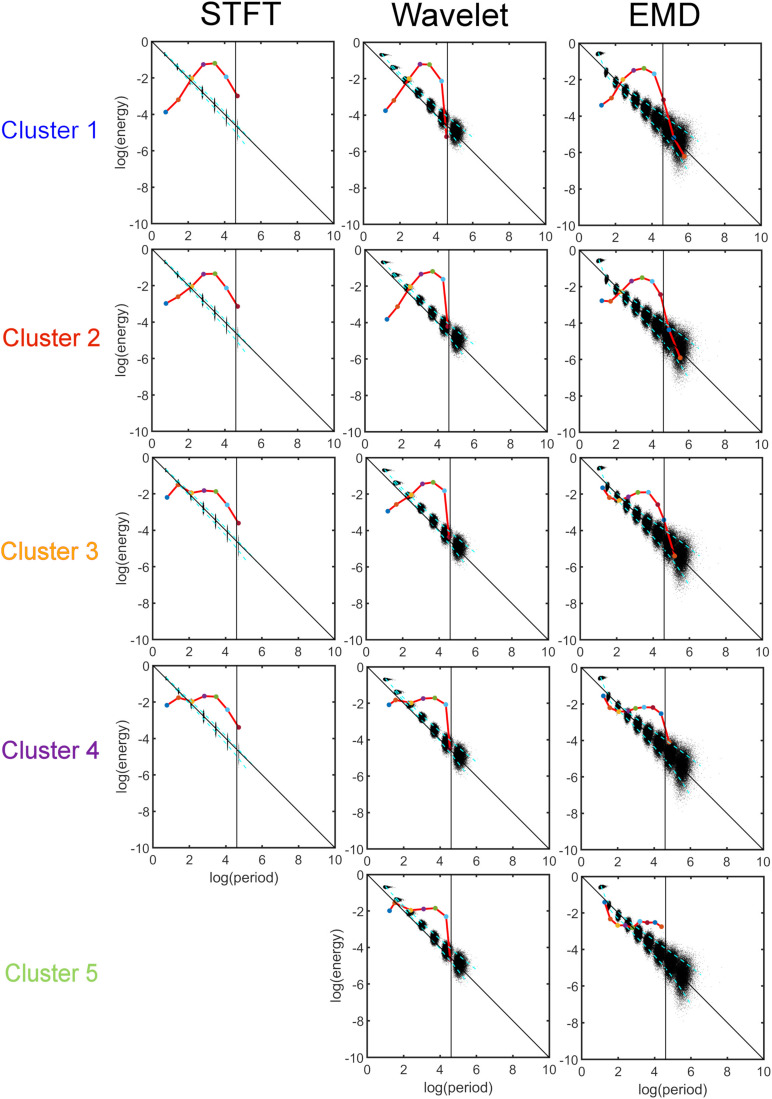
*K-means* clustering of the energy-period profiles for all 30 ICA components, where each ICA time series was decomposed into components using three different analysis methods (Short-Time Fourier Transform (STFT), Maximal Overlap Discrete Wavelet Transform (MODWT), and Empirical Mode Decomposition (EMD)). The vertical black line indicates the frequency cut-off value of 0.01 Hz, and the black dots indicate the profile of Gaussian white noise. The red line for STFT and Wavelet consists of 6 components each, whereas the redline of EMD spans 9 IMFs. Summary: Note that STFT and Wavelet use the same number (i.e., 7) of decompositions to reach the 0.01 Hz line for all clusters. EMD, however, is cluster-specific and adapts its number of IMFs to the frequency content of the clusters. Thus, EMD uses 7 IMFs to reach the 0.01 Hz line for the low-frequency cluster 1, and 9 IMFs for the high-frequency cluster 5. For clusters 2 to 4, the number of IMFs used by EMD is in-between, depending on the frequency content of the clusters.

Figure 8 shows the results of the *K-means* clustering according to the method described in Section “Clustering of Components Based on the Energy vs. Period Relationship.” The number of clusters found by applying criteria given in Eq. 10 is four for STFT, five for MODWT, and five for EMD (see Supplementary Material 2). For EMD *nine* intrinsic mode functions (IMFs) were used in the clustering, whereas for the STFT and MODWT methods *seven* detail components were suffient in the clustering because the energy of components higher than seven was essentially zero unlike EMD.

**FIGURE 9:**
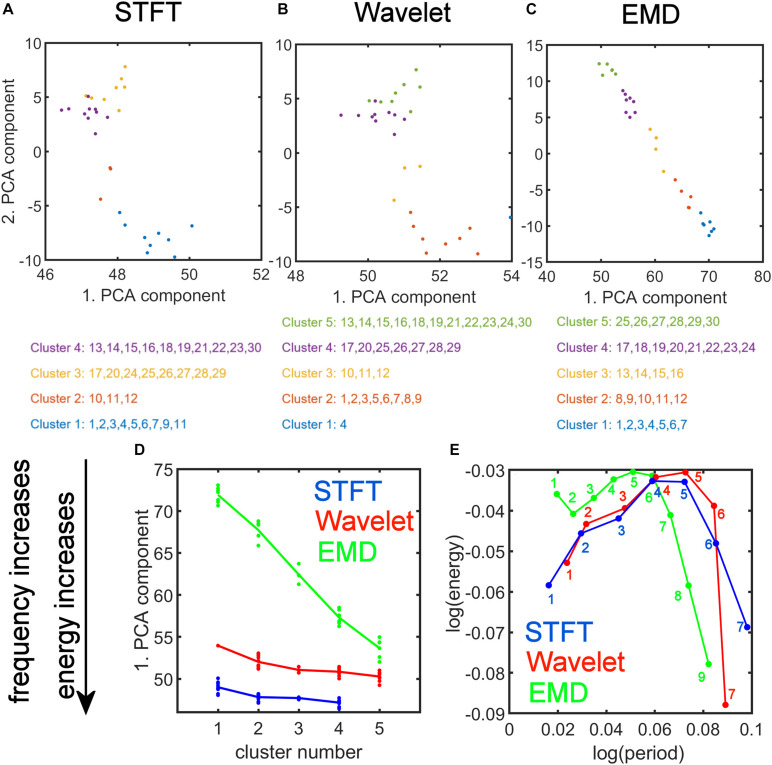
Strong Relationship of EMD to Principal Component Analysis (PCA). *Top*: (A,B,C) PCA of the feature data matrix obtained by the Short-Time Fourier Transform (STFT), the Maximal Overlap Discrete Wavelet Transform (MODWT), and Empirical Mode Decomposition (EMD). The scatter plots show the first and second PCA component values for 30 resting-state networks that were classified into four clusters by STFT **(A)**, five clusters by the MODWT **(B)**, and five clusters by EMD **(C)**. Networks belonging to the same cluster have the same color. *Bottom*: **(D)** Relationship of the value of first PCA component to frequency and energy content (see downward pointing arrow) of the clusters using different methods (STFT, MODWT, and EMD). **(E)** Feature values for log(*period*) and log(*energy*) corresponding to the first eigenvector for each decomposition level (IMFs 1–9 for EMD, 1–7 for STFT and MODWT), averaged over all subjects. Note that the contribution to the energy is largest for IMFs 1–6 of the EMD method among the three methods. Summary: Note that the clusters determined by EMD **(C)** show a strong (almost linear) relationship in the 2-dim PCA plot, which is not the case for STFT **(A)** and Wavelet **(B)**. Also note that EMD has a strong frequency and energy relationship to PCA **(D)**. As shown in panel **(D)**, EMD provides the largest separation in frequency and energy content of the clusters of ICA networks compared to STFT and Wavelet methods. In fact, clusters identified by EMD are characterized by increasing frequency and energy content with increasing cluster number (i.e., the higher the cluster number, the higher will be its frequency content), whereas the corresponding figures for STFT and Wavelet are rather flat and uninformative of the frequency content.

We plotted the transformed feature matrix $\tilde{X}$ (see Eq. 12) for the first two principal components as a 2-dimensional (2-dim) scatter plot for STFT, MODWT, and EMD, and indicated the cluster membership of each resting-state network according to the results from *K-means* clustering (Figures 9A–C). Different cluster memberships are shown in different colors. The EMD method shows that the 30 resting-state networks are found along the diagonal line and that all five clusters are well separated in this 2-dim PCA plot. We further illustrate in Figure 9C that for EMD the 1st PCA component of the data provides already a clear separation of the different clusters contrary to the STFT and MODWT methods. In Figures 9D,E, we show scatterplots of the decomposition level of the first eigenvector for each method by using Eq. 12 to obtain log(*period*) and log(*energy*) information averaged over all subjects. We also indicate the direction of frequency increase and energy increase for Figure 9D, which follows directly from the relationship of the term $\tilde{X}\,\left(:,1\right)=X\,V\,\left(:,1\right)$ to the term *V*(:,1) (first eigenvector) as shown in Figure 9E. Figure 9 most accurately illustrates the most significant differences between EMD, STFT, and MODWT.

**FIGURE 10:**
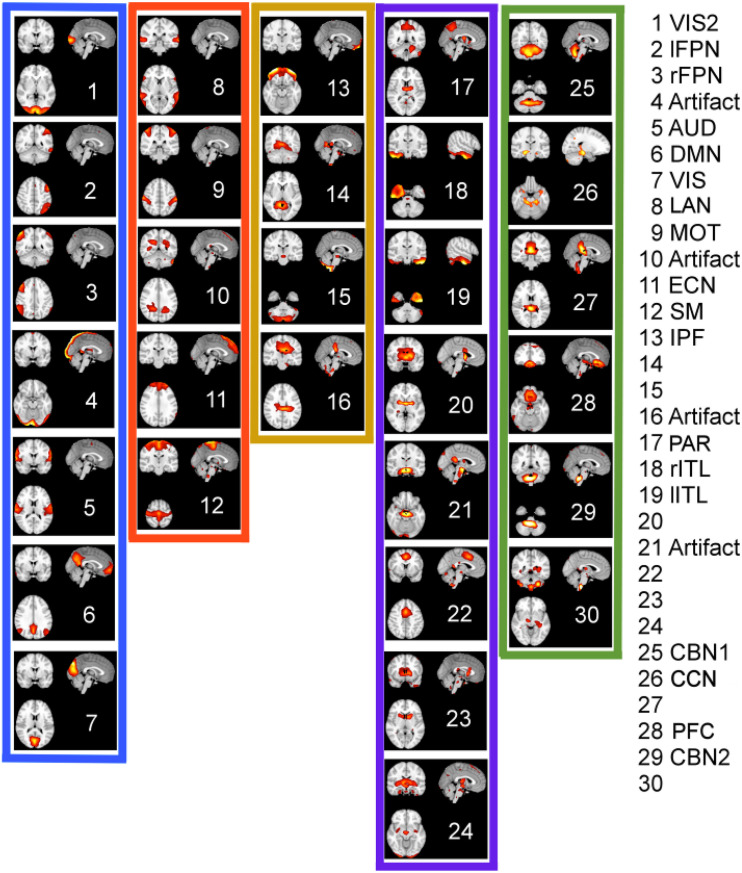
Ordered ICA components according to *K-means* clustering of the energy-period relationships of IMFs using EMD. The color-framed ICA components correspond to the five major clusters in Figures 8, 9C for the EMD method. The numbering of the ICA components from 1 to 30 was chosen by ordering the ICA components according to the value of the 1st PCA component of Figure 9C (from largest value to smallest value). Identified networks are listed on the right; a blank entry indicates that the network is unknown (to us) or has not been studied. Some of the unknown networks may be due to artifacts. Anatomical regions are listed for each network in Supplementary Material 2. Low frequency networks most studied in recent literature belong to clusters 1 and 2.

Figure 10 shows the spatial maps of the ordered ICA components according to *K-means* clustering in 2D PCA space (compare Figure 9C). The color-framed ICA components correspond to the five major clusters in Figures 8, 9C for the EMD method. The numbering of the ICA components from 1 to 30 is obtained by ordering the ICA components according to the position of the network in Figure 9C using the EMD method along the diagonal line of scatter points. Once the largest Euclidean distance of scatter points in Figure 9C is determined, the end points define ICA component #1 which is chosen as the lower right scatter point in Figure 9C, and #30 which is the upper left scatter point in Figure 9C. All other ICA components are characterized according to the Euclidean distance measured from the first data point in the direction of the last data point. Identified networks are listed on the right in Figure 10; a blank entry indicates that the network is unknown (to us) and has not been studied (to our knowledge). Some of the unknown networks may be due to artifacts and more detail on the networks is provided in Supplementary Material 3, where the major brain regions of each ICA component are listed according to the *Automatic Anatomic Labeling* (AAL) Atlas (Tzourio-Mazoyer et al., 2002). Some of the ICA components are listed as artifacts when the artifact was easy to identify. For example, ICA component #4 is most likely a motion artifact and ICA component #10 a mathematical artifact originating from the variance normalization of the original time series. Furthermore, it can be seen that only white matter regions in the interior of the brain are affected by ICA #10. ICA components #16 and #21 also involve white matter regions of the brain as well, which may indicate that these components are also artifacts.

### Clinical Application to Early-Stage Never-Medicated PD (PPMI Data)

In the Supplementary Material 6 we show the spatial maps and corresponding energy-period profiles for six resting-state networks for the three different methods (STFT, MODWT and EMD). The six studied networks are the executive control network (ECN), the parietal network (PAR), the cognitive control network (CCN), the (inferior) prefrontal cortex network (PFC), and the left/right frontoparietal networks (lFPN, rFPN). These networks were chosen based on our research on dynamic functional connectivity analysis in PD (Zhuang et al., 2018) and shown to be part of an exclusive *backbone network* in a sub-sample of PD (Mishra et al., 2020). While all of these networks show spatial and temporal differences, the most significant differences in log(*period*) between NC and PD were found by EMD. We indicated mean differences (over subjects) of |*log*(*period*)[*NC*])−*log*(*period*)[*PD*]|≥0.1 within the energy-period plots and marked significant differences (*p* < 0.05) by a star. For the STFT method, there were no differences ≥0.1 found in log(*period*) for any of the components. For the MODWT, only two networks showed differences in period (ECN with component #5 and PFC with component #2) whereas EMD showed differences in period for *all* six networks *and all IMFs with index > 1*. Furthermore, the periods of the low-frequency IMFs (index > 1) were found to be larger in PD for most networks, and the amplitude of oscillations of the IMFs as measured by the energy were found to be generally smaller in PD for many of the IMFs. We did not label significant energy differences (see Supplementary Material 6) to preserve simplicity, though energy differences are clearly visible.

**FIGURE 11:**
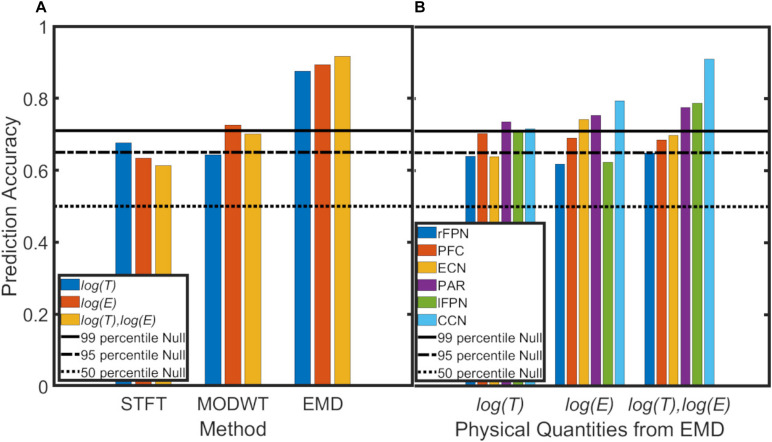
Prediction accuracy of PD vs. NC using a support vector machine and the leave-1-out method applied to 6 brain networks (ECN, PAR, CCN, PFC, lFPN, rFPN) (see Supplementary Material 6). Black line, dash-dotted line and dotted line represent the prediction accuracy of the null (permutation) distribution at the 99 percentile, 95 percentile and 50 percentiles. **(A)** Data for all 6 networks were combined. EMD (with all 5 IMFs combined) shows the highest prediction accuracy for period (log(T)) information (blue), for energy (log(E)) information, or both period and energy (log(T),log(E)) information combined. Overall, the Short-Time Fourier Transform (STFT) shows lowest prediction accuracy and the Maximal Overlap Discrete Wavelet Transform (MODWT) performs intermediate. **(B)** Network-specific prediction accuracies for EMD (with all 5 IMFs combined). Period information of all 6 networks are either very close or above the 95 percentiles of the null distribution. For energy values, only the prefrontal cortex network (PFC), executive control network (ECN), parietal network (PAR) and cognitive control network (CCN) are significant (above the 95 percentiles of the null distribution) whereas the right and left frontoparietal networks (lFPN, rFPN) do not reach significance. Combining period and energy information yields significance for all of the 6 networks.

For the machine-learning application, average prediction accuracies were determined for log(T) data, log(E) data, and both log(T) and log(E) data of all six resting-state networks combined and for each method (STFT, MODWT, EMD) (Figure 11A). Furthermore, *network-specific* prediction accuracies were computed and are shown for EMD only in Figure 11B. The null distribution of the prediction accuracy was also computed using permutation analysis, and the thresholds for the 50, 95 and 99 percentiles were determined to be *PA* = 0.50, 0.64, 0.69,respectively. Figure 11A shows clearly that EMD has the highest prediction accuracy (0.92) among the three methods using the combined resting-state networks. For EMD, the prediction accuracy is well above the 99 percentiles of the null distribution. For the *individual* networks in Figure 11B, we determined that prediction accuracies using period information are either close to or above the 95 percentiles of the null distribution, whereas for energy values only the prefrontal cortex network (PFC), executive control network (ECN), parietal network (PAR), and cognitive control network (CCN) are significant (above the 95 percentiles of the null distribution). Combining period and energy information yields significance for all of the six networks individually.

## Discussion

### Technical Aspects of Applying EMD to fMRI Data and Simulated Data

#### Advantages of Using EMD and Hilbert Transform in fMRI

EMD is essentially a sifting algorithm that estimates the local mean of a time series and subtracts it successively until criteria are satisfied that define an IMF. An IMF is defined as a basic oscillatory function, where each extremum is followed by a zero crossing. Since the IMF is oscillating and has a local mean equal to zero, the Hilbert Transform is well defined and produces meaningful non-negative instantaneous frequencies.

Unlike Fourier or wavelet transforms, neither stationarity nor linearity of a time series are assumed when using the EMD and Hilbert Transform. There are also no *a priori* defined basis functions used for EMD, rather the data are used to determine their underlying basis functions constituting the IMFs. Fixed basis functions are useful when the underlying physical process of a time series is known, which is rare for non-stationary data. EMD determines the IMFs of the data, providing a local, adaptive, data-driven description of the oscillatory components more appropriate for fMRI signals.

More specifically, the EMD method provides IMF basis functions, which are extensions of Fourier basis functions where amplitude and frequency no longer are constants: instead, amplitude and frequency are time-dependent leading to simple oscillatory functions, where each extremum is followed by a zero crossing. These characteristics allow instantaneous time-frequency information to be obtained by using the Hilbert Transform. For the MODWT transform, in contrast, the wavelet coefficients and the reconstructed multi-resolution functions do not show the features of simple oscillating functions but, rather they can have so-called riding waves where two or more adjacent extrema occur without zero crossing in between. Due to these riding waves, there is more than one simple instantaneous frequency value involved such that a computation of the instantaneous frequency value by the Hilbert Transform is only approximate at locations with riding waves.

Furthermore, in applying EMD to fMRI data, we found that IMF_1_ usually represents essentially high frequency oscillations. Of note, due to its additivity property, EMD can compute these high frequency oscillations in a *local* neighborhood and subsequently remove them from the data at a local level. This approach is quite different than, for example, low-pass Fourier filtering, because local non-stationary information is taken into consideration by EMD whereas low-pass filtering using stationary transforms removes non-stationary aspects of the signal by smoothing.

#### Computation of Average Energy and Average Period of Each IMF

After applying EMD and Hilbert Transform to fMRI data, we obtained the instantaneous period and energy of each IMF. The relationship between average energy and average period is then computed to evaluate the energy-period characteristics of each IMF. The calculation of the average energy density of the IMFs is straightforward, whereas the average period is more difficult to compute. A simple approximation exists to determine the average period *T* by the relationship

(20) $$T=\frac{l\,e\,n\,g\,t\,h\,o\,f\,s\,i\,g\,n\,a\,l}{\frac{\#\,o\,f\,z\,e\,r\,o\,c\,r\,o\,s\,s\,i\,n\,g\,s}{2}}.$$

This definition is only approximately true, however: particularly for the higher IMFs which contain larger periods, this leads to significant errors because this approximation does not take non-stationary local properties of the signal into consideration (Wu and Huang, 2004). Determining the average period via the instantaneous frequency relationship by using the Hilbert Transform is more accurate (Huang and Shen, 2014).

#### Period Characteristics Computed by EMD Show Higher Sensitivity to the Type and Amount of Correlations in Simulated Time Series When Compared to STFT and MODWT

In our AR(1) noise simulation we have shown that the lines for log(period) computed by EMD have a smaller slope and larger offsets than STFT and MODWT. Furthermore, and more importantly, the lines for log(period) vary significantly as a function of the AR(1) coefficient ϕ using EMD but not for STFT and MODWT. Since the AR(1) coefficient determines the type (i.e., positive or negative) and also strength of correlations in a time series, which in turn leads to different amounts of high and low frequencies in the data, EMD is a far more sensitive method than STFT and MODWT to detect small differences in frequency content in data.

### Energy-Period Characteristics of Resting-State Networks Are Different From Gaussian White Noise

The energy-period relationships of the IMFs for resting-state networks are quite different from Gaussian white noise properties. In general, the intermediate IMFs bulge out above the diagonal white noise line defined by *log*⁡(*E*) + *log*⁡(*T*)≈0 and signify networks with strong positive autocorrelations.

### Identifying ICA Networks With Similar Energy-Period Relationships With K-Means Clustering

Clustering of the shape of these energy-period profiles leads to well-defined ordering of ICA components. Initially, the order of the group ICA components determined from the data do not have any particular order after ICA, which is quite different than a PCA decomposition where components can be sorted based on the magnitude of their eigenvalues.

Using *K-means* clustering in IMF feature space, we obtained five major clusters and were able to characterize each network component according to the form of the energy-period relationship using the two largest PCA components. It is interesting to see that cluster 1 (with the largest profile difference compared with white noise) contains those *traditional* resting-state networks that have been most often described in the literature, whereas clusters 4 and 5 contain networks that have cerebellar and subcortical features. These clusters have a profile closer to, but still different from, the white noise line but have unfortunately generated little interest in the scientific community. Since clusters 4 and 5 contain higher frequencies (as we showed in Section “Comparison of Energy-Period Profiles for Five Common Networks that Differ in their Frequency Content,” Figure 9D) than the traditional resting-state networks and EMD provides a unique characterization of these networks (Figure 9C), the appearance of these networks as distinct clusters highlights a strength of the EMD method in characterizing resting-state networks with higher frequency content.

#### Justification of Same Feature Space in K-Means Clustering Among Different Subjects

A concern about using EMD feature space in the k-means clustering method is whether frequency and energy content of the *k*^*th*^IMF with subject 1 is comparable with frequency and energy content of the same *k*^*th*^IMF of subject 2. Conventionally, for a Fourier frequency-based analysis, the components of a feature vector are ordered according to information about frequency 1, frequency 2, frequency 3 and etc. Therefore, for a Fourier-based ordered feature vector, each component of the feature vector is associated with the same frequency value for all subjects, resulting in feature vectors where each component has a precise meaning related to the chosen frequency partitioning. In the following, we would like to point out that (1) the same associations exist for EMD based features, and (2) why an EMD-type feature vector may lead to a better description of features in the data than a Fourier-based feature vector.

First, our assumptions are that resting-state data in a group of young healthy subjects have similar brain networks which can be extracted by a group ICA analysis (which is supported by extensive literature in this area), and that EMD for data with the same underlying signal distributions has the property of being a quasi-dyadic filtering method. Consequently, the notion that the IMF_1_ of a subject could become IMF_2_ in another subject is inconsistent with similar group data. Furthermore, and perhaps most important as demonstrated in the experimental evidence given in this study, all data points for the same IMF *from all subjects* are clustered together, suggesting that *the same indexed IMF is comparable across all subjects*. For example, Figure 4C shows that all 22 subject-specific data points are very close together for IMF_1_ (22 dark blue dots), IMF_2_ (22 orange dots), IMF_3_ (22 yellow dots), etc, and all different colored points are well separated and do not overlap. This behavior is true not only for the default mode network, but for all ICA brain networks investigated in this study. Even for the higher-order noisy ICA networks that contain high frequency information, the mean period characteristics are well separated for different IMFs, and the mean periods of IMF_*k*_ never overlap with mean frequencies of IMF_*m*_ when k is unequal to m. This separation of features for different orders *k* of IMFs can be attributed to the small spread of period information among subjects. Whereas the energy spread *log*(*E*_*k*_) can be up to 2 units, the period spread *log*(*T*_*k*_/*s*) is not more than 0.2 units for IMFs 1 to 7 among the 22 subjects. Finally, the k-means clustering that we propose is a clustering in an *adaptive* feature space. The feature vector we chose is defined in the IMF index space and we cluster according to component entries of features of IMF_1_, features of IMF_2_, features of IMF_3_, etc.

#### Comparisons of Clustering Performances Among Features Derived From EMD, STFT, and MODWT

The energy and period relationships as a function of the component (detail) level corresponding to the dyadic decomposition give identical straight lines with the same slope for the MODWT and STFT methods, whereas EMD produces an adaptive curve that shows decreased periods (higher frequency content) for the IMFs with larger index *depending on the data*. The period spacing for the different detail levels is the same for STFT and approximately the same for the MODWT. However, the MODWT detail-level decomposition shows some variation in period for each subject. In contrast, the EMD method leads to an adaptive purely data-driven decomposition in period content which is also different for different brain networks. Thus, the EMD approach retains potentially important and physiologically relevant features of brain networks that are lost with STFT and MODWT.

Clustering of energy-period features in IMF feature space shows that the so-called traditional networks such as DMN and ECN of clusters 1 and 2 have characteristic bell-shaped profiles with large positive autocorrelations across decomposition levels, whereas the high-order clusters 4 and 5 show more flat profiles closer to the Gaussian noise line (Figure 5). The period as a function of the decomposition level for all clusters has the same slope for STFT and MODWT, but for EMD this slope is unlike the different clusters. Here, IMF low-frequency content is largest for traditional networks in cluster 1 (for example the DMN) and decreases as the cluster number increases (for example the cerebellar networks in cluster 5 (for example CBN1) have smallest slope in Figure 7 indicating high frequency content). Cluster 5 clearly shows that the largest contribution to the energy content is related to IMF_1_, which has high-frequency content and very likely corresponds to high-frequency physiological processes. Using the STFT and MODWT, a much lower energy content is extracted for their first high-frequency component. For all other components, the energy extraction by EMD is reversed and IMFs 2–4 have lowest energy content. Though IMFs 1 to 4 have profiles close to the white noise diagonal line, the higher-order IMFs 5 to 9 are significantly different from white noise characteristics (see profiles of CBN1). In particular, EMD leads to a *significant high-frequency shift* of these higher order IMFs, which is not the case for STFT and MODWT. For example, EMD leads to an IMF_6_ that has *log*⁡(*T*/*s*) = 3.2 whereas the corresponding decomposition using STFT and MODWT has *log*⁡(*T*/*s*)≈4.2. This shift increases with increasing IMF index. Overall, before we reach the drift frequency range (*f* < 0.01), we obtain nine decompositions using EMD instead of the six derived from STFT or MODWT. Thus, EMD shows its most adaptive behavior for cluster 5 leading to very different temporal characteristics of the IMFs than decompositions by STFT and MODWT.

#### PCA Decomposition of Energy-Period Feature Space and Relationships to K-Means Cluster Number

A low-dimensional PCA decomposition of the feature vectors is also instructive and shows that only EMD provides a significant monotone relationship of the value of the 1st PCA component as a function of the cluster number. Since all resting-state data points occupy the smallest region in PCA space (Figure 9C), the clusters are clearly separable in low-dimensional PCA space for EMD, but not for the STFT and MODWT methods. This figure suggests that EMD (among the three methods) provides a representation of the different types of resting-state networks leading to well-defined clusters. The significance of this statement is that physical quantities derived from EMD are more sensitive to features in the data, which we demonstrate in the clinical application using machine-learning (see also Section “Identifying ICA Networks with Similar Energy-Period Relationships with K-Means Clustering”). The well-defined clusters provide information of the amount of high-frequency content of the networks belonging to the clusters. For example, Figure 7 shows 5 networks that are representative of the 5 clusters (cluster 1 (DMN), cluster 2 (ECN), cluster 3 (IPF), cluster 4 (rITL), cluster 5 (CBN1)), where each IMF has increased frequency (lower period) with increasing cluster number. Thus, the networks belonging to cluster 1 are low-frequency networks without much frequency content above 0.1 Hz whereas the networks of cluster 5 have significant high-frequency content.

Furthermore, since each eigenvector of the feature matrix is linearly related to the PCA component values (from Eq. 12), a decrease of the 1st PCA component is associated with a differential increase in energy and a decrease in period. Thus, for the EMD method, larger cluster numbers are associated with networks that operate at higher frequencies and larger energy content, whereas the MODWT and STFT methods lead to weaker relationships due to the smaller slope of 1st PCA component as a function of the cluster number (as shown in Figure 9D). In particular, for a PCA component to become smaller, it can be deduced from Figure 9E that | *log*⁡(*period*)| and | *log*⁡(*energy*)| need to decrease. Since *log*⁡(*period*) > 0 and *log*⁡(*energy*) < 0, it follows due to monotonicity of the logarithmic function for *energy* > 0and *period* > 0 that *period* needs to decrease and *energy* needs to increase. *Therefore, a decrease of the first PCA component will lead to an increase in energy and a decrease in period, i.e., an increase in frequency*. Using EMD, we find that the 1st PCA component decreases monotonically with increasing cluster number (Figure 9D). *Thus, energy and frequency content increase in EMD with increasing cluster number.* This relationship is strongest for EMD, weaker for the MODWT method, and weakest for the STFT method. The scatter plot of Figure 9E shows how the energy and period are affected for each decomposition level. For example, an increase in energy is largest for IMFs 1–6 of the EMD method when increasing the cluster number. This energy increase is smaller for STFT and MODWT methods for the same decomposition levels. The sum of these findings together demonstrates that EMD has significant advantages in characterizing brain networks compared to STFT and MODWT.

### Frequency Range of Resting-State Brain Networks (Comparison to Niazy et al., 2011)

Niazy et al. (2011) used EMD to study the frequency content of the time courses associated with four major resting-state networks (DMN, VIS, AUD, MOT) for a small group of normal subjects using a TR of 3 s. EMD applied to these data gave four IMFs that covered the frequency bands (approximately) 0.004 Hz–0.01 Hz, 0.01 Hz–0.02 Hz, 0.02 Hz–0.05 Hz and 0.06 Hz–0.15 Hz. Overall, we agree with the findings from Niazy et al. and other studies (for example Boyacioglu et al., 2013) that the majority of frequency components for these four primary networks is mostly in the low frequency range below 0.1 Hz. Our analysis shows that IMFs 3–7 have significant energy and frequency content in the 0.01 Hz–0.2 Hz range for most of the traditional networks associated with clusters 1–2. For the DMN, however, the frequency range when weighted by the energy density is more limited because IMF_2_ has low energy density. We found that the DMN has peak frequency content spanning the interval of 0.02 Hz–0.09 Hz, which is covered by IMFs 3–6. However, IMF_2_ (though having far less energy density than IMF_3_) with peak frequency 0.21 Hz can produce an acceptable map of the DMN since the DICE similarity coefficient is 0.68 [see spatial IMF maps and computation of the DICE similarity coefficient (Figure 4D and Supplementary Material 1)]. On the contrary, IMF_1_ contains high-frequency noise sources that are not related to features of the DMN. Taken together, these findings indicate that the DMN is characterized mostly by low frequency information and frequencies much larger than 0.2 Hz will not yield maps that resemble the DMN. For other recent applications of EMD in neuroscience, please see the Supplementary Material 4.

### EMD-Derived Features Contain More Clinical Meaningful Information

We applied the proposed frequency analysis methods to resting-state fMRI data from PD subjects. Energy-period characteristics profiles have been derived using EMD (the proposed method), STFT and MODWT. Performances of these features in bringing clinically meaningful information are compared. In PD, changes in whole-brain functional connectivity have been recently observed affecting *wide-spread cortical* regions. For example, Tang and Eidelberg described a so-called *PD-related cognitive pattern* (Eidelberg, 2012) and de Schipper et al. (2018) found PD-related changes in resting-state functional connectivity in frontoparietal brain regions. In another study involving motor and depressive symptoms in PD, Song et al. (2015) found unique associations of fMRI band signals obtained with EMD in specific cortical and subcortical brain regions. Qian et al. (2017) investigated frequency-specific brain networks in PD with and without depression and found significantly disrupted nodal topological characteristics (reduced regional efficiency) in frequency bands 0.02 to 0.05 Hz, spanned by IMF_3_ (for data acquired at TR = 2 s) in the visual association cortex in the non-depressed PD group. We demonstrated that in early-stage, never-medicated PD there are significant differences in the temporal and energy characteristics of several traditional resting-state networks when EMD is applied, which cannot be found with STFT and MODWT. Specifically, using STFT, MODWT, and EMD, we have analyzed the first five components (decomposition levels) of the ECN, PAR, CCN, PFC, lFPN, and rFPN networks in terms of their average energy and average period content and found that the EMD method provided the most significant differences between PD and NC among the three methods studied. Most of the obtained EMD IMFs in PD for PAR, CCN, PFC, lFPN and rFPN showed oscillations with significant increased period (decreased frequency) and decreased energy content. Importantly, despite a lack of *a priori* modeling or analytic assumption, the data-driven EMD approach demonstrates a reduction in network frequency and energy in PD consistent with our hypothesis, and which is consistent with (but expands upon) other imaging and electrophysiological studies (Mazzoni et al., 2012; Prashanth and Strafella, 2012; Cagnan et al., 2015). We have obtained consistent features of energy and period for all subjects using EMD, as shown by the increased difference in mean values and the small standard deviations about the mean values. Though all three analysis methods share a frequency decomposition feature exhibiting an exact or quasi dyadic filterbank decomposition, only EMD provides a model-free, adaptive, and entirely data-driven method to decompose time series. The computed prediction accuracies using machine learning provide evidence that EMD is a superior time series analysis method to classify NC and PD subjects based on energy-period relationships, which cannot be achieved using Fourier and wavelet-based methods.

### Limitations and Future Directions

#### General Limitations

For noisy time series with limited duration, the EMD method could yield non-orthogonal IMFs that give rise to a covariance matrix with significant non-diagonal contributions. These effects occur only for the higher IMFs that represent very low frequencies, however, and are seen when the number of cycles of these very low frequencies are not sufficiently covered by the length of the data. In our case, this effect is not an issue because we high-pass filtered the data to eliminate frequency processes less than 0.01 Hz and our data length (30 min resting-state data) covered a large number of periods for the lowest possible frequency considered (period T = 100 s). However, for other data lengths of only a few minutes (not an uncommon duration in fMRI literature) the computation of the higher IMFs (8, 9) may lead to non-orthogonal components. Thus, resting-state data with a duration of sufficient length (about 10 min or larger) should be collected to prevent problems of non-orthogonality of the higher IMFs if very low frequency information is to be investigated.

A limitation of EMD is that the IMFs obtained are specific to the sampling rate (TR in fMRI). Thus, the IMFs do not represent TR-independent oscillations of the brain but are more related to a quasi-dyadic bandpass filtering decomposition. Thus, IMFs for data obtained with a TR = 2 s will usually not correspond to any IMFs for data obtained at a TR = 0.765 ms, even if the data come from the same source or subject. For example, the mean log periods (log(*T*_*k*_)) obtained for Gaussian white noise IMFs (indices *k* = 1:7) sampled at TR = 0.765 ms or TR = 2.0 s yield {1.06,1.48,2.01,2.54,3.09,3.67,4.27} and {2.02,2.45,2.97,3.50,4.06,4.63,5.23}, respectively. These numbers are clearly different. Since the underlying process is *white* noise, these numbers, however, are still equivalent and can be converted to unit sampling frequency (1 Hz) by simply subtracting the value *log*⁡(*TR*) resulting in the same exact values given by {1.33,1.75,2.28,2.81,3.36,3.93,4.53}. For real data, though, there is no correspondence because of frequency aliasing which depends on the TR. Even when frequency aliasing does not occur as in low-pass filtering operation on data, obtained IMFs are different compared to the unfiltered data. For example, the mean log periods obtained for the DMN network IMFs for the Boulder data *before* and *after* low-pass filtering with cut-off frequency 0.25 Hz using a continuous wavelet filter (time-bandwidth parameter = 50 of the Morse wavelet with symmetry parameter gamma = 3) yield for the first few IMFs the values {1.21,1.70,2.37,2.97,3.58,4.15,4.65} and {1.96,2.58,3.17,3.85,4.38,4.85}, respectively. Note that none of the log period values obtained *before* low-pass filtering is identical or close to any of the values obtained *after* low-pass filtering even though only high frequencies above 0.25 Hz have been removed from the data and the entire low-frequency spectrum is the same. Thus, IMFs for wide-spectrum data with different upper frequency value (0.65 Hz vs. 0.25 Hz) do not represent *characteristic TR-independent* frequency components of brain networks in fMRI but are more related to a (quasi) TR-dependent dyadic decomposition. However, as we have shown in Figure 7, the frequency decomposition by EMD is not exactly dyadic but adaptive and depends on the frequency content of the specific network time series in question. This behavior is quite different from temporal ICA since independent components representing *low-frequency* networks are not related to the sampling rate or a low-pass filtering operation on the data (if the cut-off frequency is large enough such as 0.25 Hz).

In this study we used only static variables, namely the *mean* value of energy and period derived over instantaneous time-dependent quantities. Thus, this study does not address fMRI dynamics occurring in resting-state data. However, EMD shows also promise in dynamic variables in fMRI, for example computation of optimal instantaneous window sizes in *dynamic functional connectivity* analysis, as we have shown recently (Cordes et al., 2018).

#### Artifacts Introduced by EMD

At endpoints and gaps in the data, EMD produces artifacts because spline interpolation is not possible due to missing local neighborhood information of data points. In general, higher order IMFs with larger periods are more affected by missing data or endpoints. This effect, however, can be easily mitigated by adding data in the reverse order. This technique is called *data mirroring* and provides local neighborhood information so that EMD does not find singularities at these points. After the IMFs are obtained, the mirrored data segments are removed from the IMFs. We excluded these endpoint artifacts in the data.

Another potential problem leading to artifacts is the so-called *mode mixing.* In our analysis, we have not observed mode mixing in our ICA time series. For more information on this topic, please see Supplementary Material 5.

#### Future Directions

Analysis of motion artifacts in the data revealed that the average motion in the young healthy cohort data was small (root mean square (rms) motion 0.6 mm or less). Due to the relatively-small motion and, more importantly, to avoid contaminating the data by introducing high-frequencies by common regression approaches (Chen et al., 2017), we have not carried out any motion correction in this study except the initial realignment. Furthermore, correction for physiological (heart rate and respiratory rate) noise or global signal regression was not performed to keep the preprocessing steps as simple as possible. However, how the EMD results are affected by motion regression and by physiological noise correction are important in the analysis of resting-state data and will be further investigated in future studies.

## Conclusion

We have studied resting-state networks using EMD to obtain instantaneous time-frequency-energy information. IMFs and associated spatial maps provide a data-driven decomposition of resting-state networks, free from a priori assumptions or modeling. We investigated the average energy-period relationship of IMFs of group ICA networks to better characterize temporal properties of networks and found that the IMFs of BOLD data provide characteristic energy-period profiles that allow a data-driven arrangement of all resting-state networks when compared to profiles of pure noise. Such an arrangement is not possible using the STFT and MODWT methods, which are non-adaptive decomposition methods, although both methods provide a dyadic frequency decomposition similar (but not identical) to EMD.

Focusing our temporal analysis on *traditional* functional networks (clusters 1 and 2 in Figure 9C) showed that mostly low frequencies in the 0.02–0.06 Hz range similar to many other studies (for example, see: Achard et al., 2006) contributed to the networks, and none of these networks could be associated with any significant high-frequency content. For the DMN, the largest supported frequency was 0.21 Hz. Along with these traditional networks, we also found high-frequency networks (clusters 3,4,5 in Figure 9C) which had a significant energy content above 0.1 Hz up to the Nyquist frequency. These networks have been rarely studied, some may be related to physiological noise or represent artifacts, and only sparse information is available about their function in the literature and further studies are warranted in this regard.

In a clinical application to early PD, we used EMD to study the energy and period content of IMFs for typical resting-state networks. Compared to STFT and MODWT, EMD showed the largest differences between PD and NC subjects. Furthermore, most IMFs of the PAR, CCN, PFC, lFPN, and rFPN resting-state networks were found to have decreased frequency (increased period) and reduced energy content in PD (compared to NC) as hypothesized. Using a support-vector machine classifier showed that EMD achieves highest prediction accuracies. Obtained results expand the current understanding of network dynamics in PD, and further studies are planned to investigate network dynamics and energy-period profiles correlated with clinical phenotype, disease progression, and response to treatment in PD. Energy-period relationships using EMD represent a novel approach to understanding functional networks in PD, which in turn could lead to development of a clinically useful *in vivo* assay of PD network physiology which is urgently needed in this field.

## Data Availability Statement

The raw data supporting the conclusions of this article will be made available by the authors, without undue reservation.

## Ethics Statement

The studies involving human participants were reviewed and approved by The University of Colorado-Boulder IRB committee approved the study protocol under IRB 13-0034. The patients/participants provided their written informed consent to participate in this study.

## Author Contributions

DC: conceptualization, methodology, validation, writing, and programming. MK: methodology, validation, and editing. ZY: methodology, validation, and editing. XZ: methodology, validation, and editing. TC, KS, VM, and RW: resources and editing. RN: methodology and statistics. All authors contributed to the article and approved the submitted version.

## Conflict of Interest

The authors declare that the research was conducted in the absence of any commercial or financial relationships that could be construed as a potential conflict of interest.

## Footnotes

1 www.mathworks.com

2 www.fil.ion.ucl.ac.uk/spm/

3 fsl.fmrib.ox.ac.uk/fsl/fslwiki/topup/

4 stnava.github.io/ANTs/

5 perso.ens-lyon.fr/patrick.flandrin/emd.html

6 http://www.fil.ion.ucl.ac.uk/spm/
