# Supplementary material for: Energy-Period Profiles of Brain Networks in Group fMRI Resting-State Data: A Comparison of Empirical Mode Decomposition With the Short-Time Fourier Transform and the Discrete Wavelet Transform
Source: Front Neurosci. 2021 May 21;15:663403. doi: 10.3389/fnins.2021.663403 (PMC8175789; doi:10.3389/fnins.2021.663403)
Supplement: Supplementary file 1 [file Data_Sheet_1.pdf]

APPENDIX A

A1. *Basic EMD Algorithm Steps for Obtaining the IMF Decomposition of a Signal x(t).*

0. Set $k = 0$ and $r(t) := x(t)$.
1. Find the local minima and maxima of $x(t)$.
2. Determine the lower and upper envelopes $e_l(t)$ and $e_u(t)$ by connecting respectively the minima and the maxima using cubic spline interpolation.
3. Calculate the mean $m(t) = 0.5\,(e_l(t) + e_u(t))$.
4. Extract $h(t) \coloneqq x(t) - m(t)$. If $h(t)$ satisfies the conditions for an IMF, $k \coloneqq k + 1$ and $f_k(t) = h(t)$, continue with step 5. Else $x(t) := h(t)$ and continue with step 1.
5. Set $r(t) \coloneqq r(t) - f_k(t)$. If $r(t)$ is monotone, set $k = K$ and the algorithm terminates. Else, $x(t) := r(t)$ and continue with step 1.

The iterative process sequentially explores the natural constitutive scales of a signal. The IMF with index 1 (IMF_1) contains the highest frequencies, and IMF with index K (IMF_K) contains the lowest frequency components. It has been shown that the frequency arrangement in IMFs mimics that of a dyadic filterbank (Wu and Huang, 2010).

A2. *Instantaneous Frequency and Amplitude of IMFs*

Instantaneous frequency and amplitude of the IMFs can be computed by extending the signal into the complex plane with the Hilbert transform

$$y(t) = \mathcal{H}\big(x(t)\big) = \frac{1}{\pi} PV \int_{-\infty}^{\infty} \frac{x(t)}{t - \tau} d\tau \qquad (A1)$$

where PV indicates the Principal Value of the singular integral, and $x(t)$ is the time signal of an IMF. The complex-valued signal $z(t)$ is then defined by $z(t) = x(t) + i\,y(t)$. Amplitude and phase of z are obtained by writing $z = ae^{i\phi}$. The instantaneous amplitude, $a(t)$, and frequency function, $\nu(t)$, are then calculated by

$$a(t) = \sqrt{x^2(t) + y^2(t)} \qquad (A2)$$

and

$$\nu(t) = \frac{1}{2\pi} \frac{d\phi(t)}{dt}\ , \qquad (A3)$$

respectively. It follows that the original signal can be reconstructed from the IMFs (indexed by $k = 1{:}K$) using

$$s(t) = Real\left(\sum_{k=1}^{K} a_k(t)\, e^{i\int_0^t 2\pi\nu_k(\tau)d\tau}\right). \qquad (A4)$$

In the literature, EMD combined with the Hilbert transform is referred to as the Hilbert Huang Transform (HHT). In MATLAB, there exist convenient functions to compute EMD and the Hilbert transform.

The frequency-time distribution $H(\nu, t)$ of an IMF is obtained from the function $\nu(t)$ using kernel density estimation (available in MATLAB). The frequency spectrum (also called Hilbert spectrum) is given

by

$$h(\nu) = \int_0^T H(\nu, t)dt \qquad (A5)$$

where T is the time length of the signal. Equation (A4) represents a *generalized* Fourier transform with time-dependent amplitude $a_k(t)$ and frequency $\nu_k(t)$. However, there is an important distinction between characteristics of the Fourier (or wavelet) transform and the HHT: The HHT is *not* bound by a time-frequency uncertainty relationship (Huang et al., 1998). In this regard, the HHT provides better time-frequency localization than both ordinary Fourier and wavelet transforms.

A3. *Illustration: EMD and IMFs for Nonstationary Processes*

In a simulation (see Fig.A1), we created a nonstationary signal containing an amplitude function and 3 frequency functions that change as a function of time. Explicitly, the signal is given by $s(t) = A(t)(\sin\phi_1(t) + \sin\phi_2(t) + \sin\phi_3(t))$ with amplitude function $A(t) = \sin(\omega_0\, t)$ and phase functions $\phi_1(t) = \frac{0.5}{\omega_1} sin\,(\omega_1 t) + t,\ \phi_2(t) = \frac{0.3}{2\omega_1} sin\,(\omega_1 t) + 0.6t,\ \phi_3(t) = 200\,\mathrm{erf}\left(0.004\left(t - \frac{T}{2}\right)\right) + 0.1\,t,$ respectively, with constants $T = 3000s,\ \omega_0 = \frac{4\pi}{T}$ and $\omega_1 = \frac{2\pi}{T}$. Applying the HHT to the signal produces 3 IMFs and corresponding instantaneous frequency and amplitude functions. The first IMF (IMF_1) contains the highest frequency in the signal at every time point. Similarly, IMF_2 contains the next highest (medium) frequency, and IMF_3 the lowest frequency. Discontinuities in the IMFs appear when the amplitude function is zero and when discontinuities in the *ordered* frequency spectrum arise because different frequency components have the same frequency value at a time point even though the original time series has no discontinuities (except at the beginning and end of the time series). At time points $t = \{1218, 1708\}$ the high frequency (blue curve) and medium frequency (red curve) intersect. Similarly, at $t = \{1291, 1782\}$ the medium frequency (red curve) and low frequency (yellow curve) intersect, leading to spike artifacts. Thus, EMD is not a technique that will lead to a recovery of original signals from mixed signals as ICA does. EMD only leads to a quasi-dyadic frequency decomposition. Thus, as long as the frequencies of different signals fall into different dyadic frequency bands, EMD can separate these signals. However, if at certain time points different signals have the same frequency, EMD cannot distinguish the signals and an artifact appears. This artifact is a form of so-called *mode mixing* and is a shortcoming of the EMD approach (Fosso, 2019). Also, this artifact is not related to linear dependencies in the data. We have provided more information on mode mixing in the supplementary material under point S5.

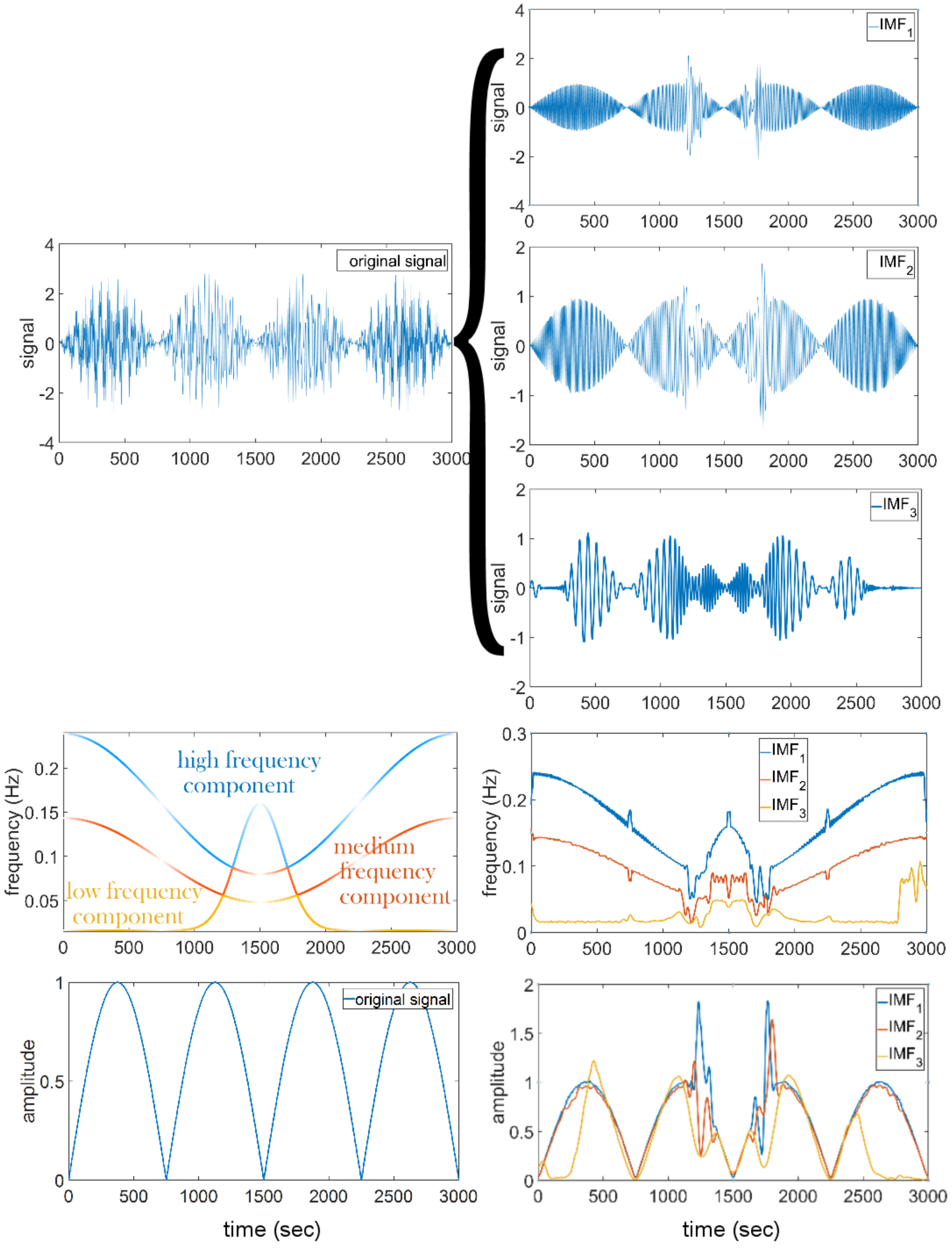

Fig.A1. EMD of a nonstationary signal given by $s(t) = A(t)(\sin\phi_1(t) + \sin\phi_2(t) + \sin\phi_3(t))$ with amplitude function $A(t) = \sin(\omega_0\, t)$ and phase functions $\phi_1(t) = \frac{0.5}{\omega_1} sin\,(\omega_1 t) + t$, $\phi_2(t) = \frac{0.3}{2\omega_1} sin\,(\omega_1 t) + 0.6t$, $\phi_3(t) = 200\,\mathrm{erf}\left(0.004\left(t - \frac{T}{2}\right)\right) + 0.1\,t$ with constants $T = 3000s$, $\omega_0 = \frac{4\pi}{T}$, $\omega_1 = \frac{2\pi}{T}$.

Top left: Original signal. Top right: First 3 intrinsic mode functions (IMFs). Middle: Time-frequency spectra of original signal (left) and IMFs (right). Bottom: Amplitude functions of original signal (left) and of IMFs (right). Note some spike artifacts of the IMFs in the frequency and amplitude spectrum.

A4. *Simulation: Instantaneous Frequencies of a Signal using EMD and the Discrete Wavelet Transform*

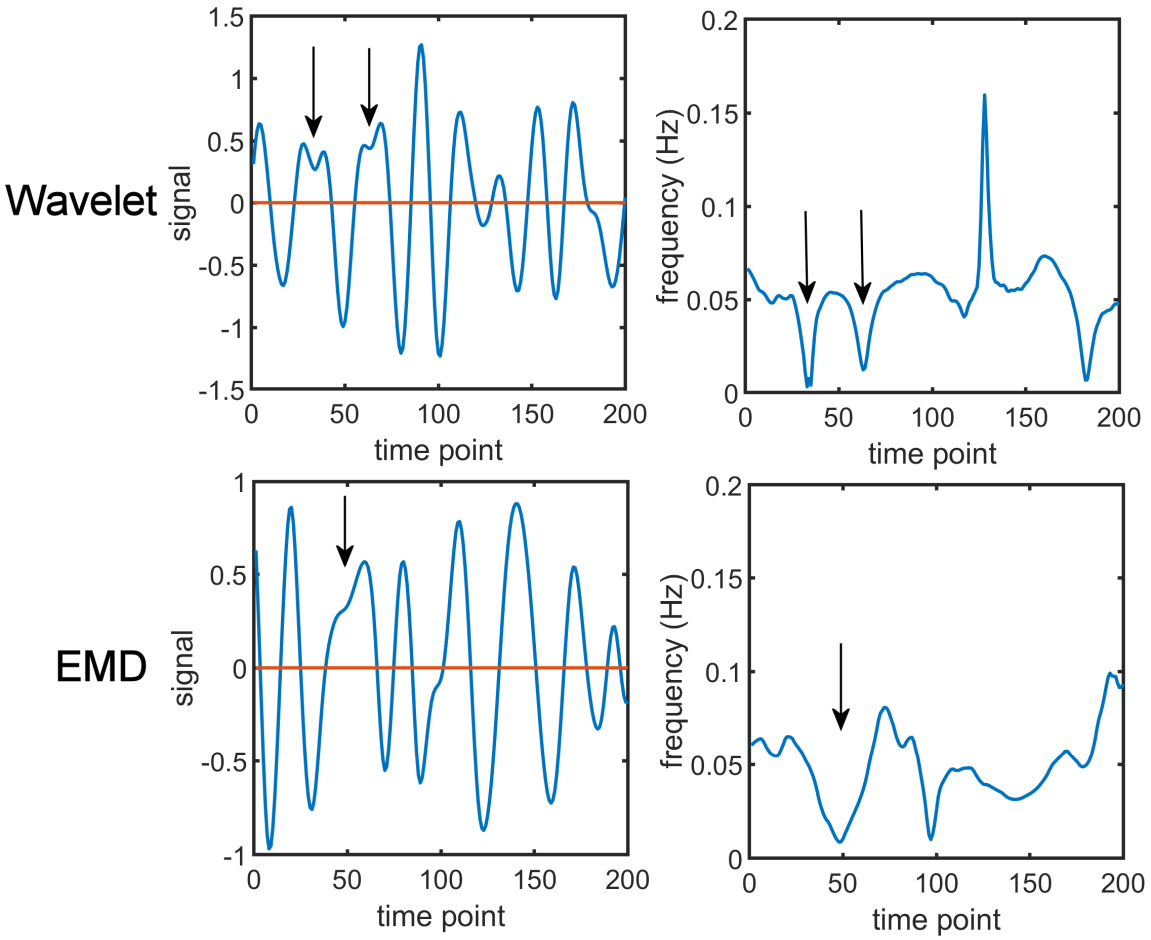

Fig.A2. Instantaneous frequencies obtained by the Hilbert transform for a maximal overlap discrete (*db6*) wavelet-transformed (MODWT) signal (top figure) and for an intrinsic mode function (IMF) using EMD (bottom figure). The wavelet coefficients and the IMF correspond to the same dyadic frequency band of arbitrary segments of a fMRI resting-state signal. The wavelet transform yields coefficients showing riding waves at specific time points (see arrows in top figure) whereas an IMF does not have riding waves.

References

Fosso, O. B. 2019. Mode mixing separation in empirical mode decomposition of signals with spectral proximity. https://arxiv.org/pdf/1709.05547.pdf.

Huang, N.E., Shen, Z., Long, S.R., Wu, M.C., Shih, H.H., Zheng, Q., Yen, N.-C., Tung, C.C., Liu, H.H. 1998. The empirical mode decomposition and the Hilbert spectrum for nonlinear and non-stationary time series analysis. Proc. R. Soc. Lond. A 454, 903-995.

Wu, Z. and Huang, N.E. 2010. On the filtering properties of the empirical mode decomposition. Advances in Adaptive Data Analysis 2(4):397-414.
