# Supplementary material for: Energy-Period Profiles of Brain Networks in Group fMRI Resting-State Data: A Comparison of Empirical Mode Decomposition With the Short-Time Fourier Transform and the Discrete Wavelet Transform
Source: Front Neurosci. 2021 May 21;15:663403. doi: 10.3389/fnins.2021.663403 (PMC8175789; doi:10.3389/fnins.2021.663403)
Supplement: Supplementary file 2 [file Data_Sheet_2.pdf]

SUPPLEMENTARY MATERIAL

Suppl. S1. Peak frequency, full-width-at-half-maximum (FWHM), frequency range, mean energy density, and period of the first nine intrinsic mode functions (IMFs) for the default mode network (DMN).

IMF #	9	8	7	6	5	4	3	2	1
Maximum Peak (Hz)	0.0031	0.0051	0.0105	0.016	0.031	0.053	0.091	0.21	0.63
FWHM (Hz)	0.0012-0.0036	0.0042-0.0074	0.0066-0.0127	0.010-0.022	0.016-0.044	0.032-0.079	0.055-0.148	0.10-0.31	0.20-0.65
Range (Hz) using 10% max	0.0010-0.0054	0.0024-0.0103	0.0035-0.0156	0.006-0.032	0.009-0.060	0.017-0.011	0.029-0.208	0.05-0.42	0.06-0.65
Mean log(energy density)	-5.80 ±0.50	-4.73 ±0.62	-2.37 ±0.43	-1.18 ±0.29	-1.07 ±0.25	-1.27 ±0.23	-1.89 ±0.24	-3.09 ±0.36	-3.31 ±0.56
Mean log(period/s)	5.72 ±0.20	5.23 ±0.13	4.65 ±0.09	4.15 ±0.09	3.58 ±0.09	2.97 ±0.10	2.37 ±0.08	1.70 ±0.07	1.21 ±0.10
Spatial similarity to group ICA map (Dice coefficient)	0.10	0.33	0.62	0.60	0.75	0.76	0.80	0.68	0.32

Suppl. S2. Cross-validation error (CVE) as a function of the number of centroids (*k*) for EMD.

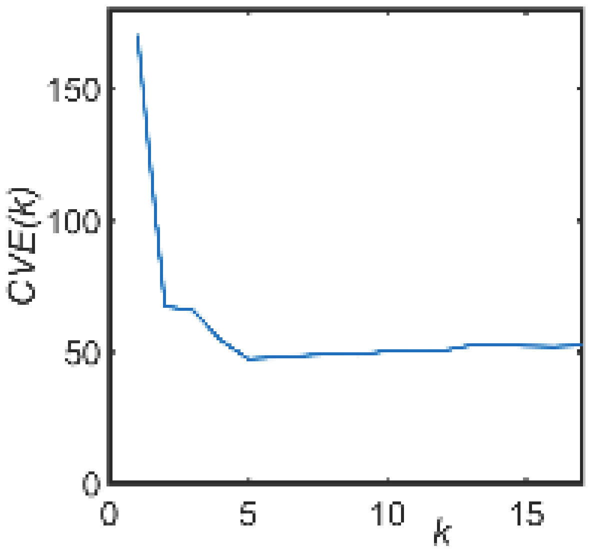

Suppl. S3. Arranged ICA components (using EMD) and brain regions involved.

ICA #	Label	Network Name	Brain Regions according to AAL Atlas
1	VIS2	Visual Association Cortex	Calcarine, Occipital_Inf, Occipital_mid
2	lFPN	left Fronto-Parietal	Temporal_Inf, Temporal_Mid, Fontal_Inf_Orb
3	rFPN	right Fronto-Parietal	Frontal_Mid, Parietal_Inf
4	Artifact	(head motion)	
5	AUD	Auditory	Heschl, Postcentral, Temporal_Sup
6	DMN	Default Mode	Cingulum_Post, Precuneus, Angular, Frontal_Mid_Orb
7	VIS	Primary Visual Cortex	Calcarine, Cuneus
8	LAN	Language	Temporal_Mid, Frontal_Inf_Orb
9	MOT	Motor	SupraMarginal, Postcentral
10	Artifact		
11	ECN	Executive Control	Frontal_Sup, Frontal_Sup_Medial
12	SM	Sensory_Motor	Supp_Motor_Area, Precentral, Paracentral_Lobule
13	IPF	Inferior Prefrontal	Frontal_Inf_Orb, Frontal_Mid_Orb, Frontal_Inf_Tri
14			Lingual, Fusiform
15			Cerebellum_8, Cerebellum_Crus2
16	Artifact		
17	PAR	Parietal	Precuneus, Caudate, Thalamus, Pallidum, Cerebellum_8, Parahippocampal
18	rITL	right Inferior Temporal	Temporal_Inf
19	lITL	left Inferior Temporal	Temporal_Inf
20			Caudate, Putamen
21	Artifact		
22			Supp_Motor_Area, Cingulum_Mid
23			Caudate
24			Thalamus, Putamen, Parahippocampus, Cerebellum_6
25	CBN1	Cerebellar 1	Cerebellum_9, Vermis_4_5_6
26	CCN	Cognitive Control	Hippocampus, Amygdala_R, Parahippocampus, Lingual, Temporal_Pole_Sup_L, Fusiform, Cerebellum_Crus1_L
27			Cingulum_Mid, Cingulum_Post, Thalamus, Cerebellum_9
28	PFC	Inf Prefrontal Cortex	Cingulum_Ant, Caudate, Frontal_Mid_Orb, Olfactory
29	CBN2	Cerebellar 2	Cerebellum_4_5_9, Vermis_10
30			Thalamus, Cerebellum_6, Cerebellum_Crus1

Note: Blank entries are unknown networks that have not been reported or investigated in the literature.

Suppl. S4. *Other Applications of EMD for Resting-State fMRI*

S4.1 Detrending

IMFs are also useful for *local* de-trending of time series. For example, the IMF_ks for k > 9 have frequencies less than 0.01 Hz (using our acquisition protocol). The local trend $\delta(t)$ of data $y(t)$ can be computed by $\delta(t) = y(t) - \sum_{k=1}^{9} \mathrm{IMF}_k$ and is certainly more accurate than a trend estimated by Fourier or other stationary filtering methods, as was recently shown (Kaleem and Cordes, 2016). However, using EMD to estimate IMFs with frequencies less than 0.01Hz is more involved because different voxel time series may have frequency components *less than 0.01Hz decomposed into IMFs with different indices*. This makes detrending with EMD more complicated than a simple high-pass filtering using regression with cosine basis functions (as we have done in this study). Alternatively, the continuous wavelet transform could also be used for high-pass filtering with cut-off frequency of 0.01Hz. We refrained from studying these very low drift frequencies with EMD or the continuous wavelet transform in this project and defer to such a study in a future application.

S4.2 Global Signal Regression

Conventionally, global signal regression uses the average of whole-brain time series as a nuisance regressor in a linear model to remove the effect of the global signal. However, this regression method does not allow for spatial variations of the global signal and treats every voxel the same, i.e. every voxel has the same regressor. Moradi et al. (2019) recently applied a spatially-adaptive EMD approach called Fast and Adaptive Tridimensional (3D) EMD (FATEMD) (Riffi et al., 2015) for removing the global signal by decomposing the fMRI data first into 5 spatial IMFs (SIMFs) and associating the spatial components SIMFs with indices 3-5 as spatially adaptive global signal masks. Then, by summing up the maps SIMF_1 and SIMF_2, the global signal can be excluded from the data.

S4.3 Single-Scale Time-Dependent (SSTD) Window-Sizes in Sliding-Window Dynamic Functional Connectivity (dFC) Analysis

In sliding-window dFC analysis, a fixed window-size is usually used and heuristically selected since no consensus exists yet on the choice of the optimal window-size. Recently, Zhuang et al. (2020) proposed the SSTD window-sizes computed from all EMD IMFs as optimal window-sizes in the sliding-window dFC analysis and applied this method to a large group of professional fighters with and without cognitive impairment, and healthy controls. Specifically, at every time point, an SSTD window-size is computed as the average of the instantaneous period over all IMFs weighted by the corresponding energy. In this case, SSTD window-sizes are based on the frequency content at every time point and are able to capture more temporal dynamic information. Both a higher classification accuracy in predicting cognitive impairment status in fighters and a larger explained behavioral variance in normal controls were found when using dynamic FC matrices computed with SSTD window-sizes as features, as compared to using dynamic FC matrices computed with conventional fixed window-sizes.

S4.4 Analytic Extension of the EMD Method

Variational Mode Decomposition (VMD) is an analytic extension of EMD (Dragomiretskiy and Zosso, 2014). VMD can be thought of as a more formal EMD-type decomposition technique, but as with all such techniques that utilize such variations of mathematical formulation of EMD-type behavior, certain parameters always must be adjusted which in our view defeats the purpose of a completely model-free and data-adaptive decomposition method. Resting-state fMRI applications have been investigated with VMD by Yuen et al. (2019) for TR=2s data and for TR=323ms data after low-pass filtering with cutoff 0.25Hz so that IMFs can be compared for different TRs. Reproducibility of IMFs and corresponding frequency clusters were found across

fMRI trials. Similar to Song et al. (2014) and our studies, Yuen et al. (2019) found cortical resting-state networks to be best represented by low frequency oscillations. VMD has also been used to avoid potential mode mixings (Yuen et al., 2019). A comparison of VMD with EMD is beyond the scope of the current study.

Suppl.S5. *Mode Mixing in EMD and Applying EMD to ICA time series*

Mode mixing in EMD refers to an intermittent signal, for example high frequency oscillations that occur in a limited time segment of otherwise low-frequency oscillations throughout the IMF. An IMF that shows mode mixing gives rise to significant non-orthogonality to the other IMFs. Mode mixings have been reported in task-activated data (Lin et al., 2016), but in resting-state fMRI data, mode mixing may not be easily observable, since no a priori knowledge of the correct signal is known. In our analysis, we have not observed mode mixing in our ICA time series. Without ICA, however, EMD can be more difficult to apply directly because of possible *mode mixings* that may occur for those voxel time series in which more than one network is active (Fosso (2019), Lin et al. (2016)). For example, if a voxel time series contains signals belonging to two different networks that operate at different frequencies in a dynamic fashion, a decomposition by EMD at a specific time point t_1 could result in an IMF order that is different for different time points (for example $\mathrm{IMF}_3\ (t_1)$ becomes $\mathrm{IMF}_4\ (t_2)$).

Without ICA, applying EMD (or related methods) directly on resting-state fMRI data can be difficult for the purpose of extracting meaningful intrinsic components and it has been speculated that the energy-period distributions would be close to white noise distributions (Lin et al., 2016). In this scenario, Ensemble EMD (EEMD) and related methods (complementary EEMD) can lead to improvements in computing IMFs by avoiding mode mixings (Qian et al., 2015, 2018). Using EEMD, noise is injected (added) into the data and multiple EMD runs are carried out for different injected noise realizations. The IMFs obtained from each run are ensemble averaged to eliminate the effect of noise. It has been shown that for a large number of noise realizations (≅60 or more), robust IMFs can be obtained that are more orthogonal to each other. Non-orthogonal contributions are evidence of under-sampled processes, thus, if non-orthogonal IMFs are found, the data segment may be too small and EEMD may provide a better estimation of IMFs. EEMD is computationally more expensive than EMD, however and furthermore, the amount of noise that should be injected for fMRI data is unknown and may have to be estimated by applying different models to the data. As a consequence, EEMD cannot be categorized as a model-free method (for detailed information on EEMD, see Huang and Shen (2014)). For completeness, we would like to mention that EEMD has been further improved by Complementary EEMD (CEEMD) (Yeh et al., 2010), then by CEEMD with Adaptive Noise (CEEMDAN) (Torres et al., 2011) and later on by Improved Complementary EEMD with Adaptive Noise (ICEEMDAN) (Colominas et al., 2014; Moradi et al., 2019). A comparison of these advanced EMD methods with our method is beyond the scope of the current study.

Suppl.S6. *Application to Parkinson's Disease*

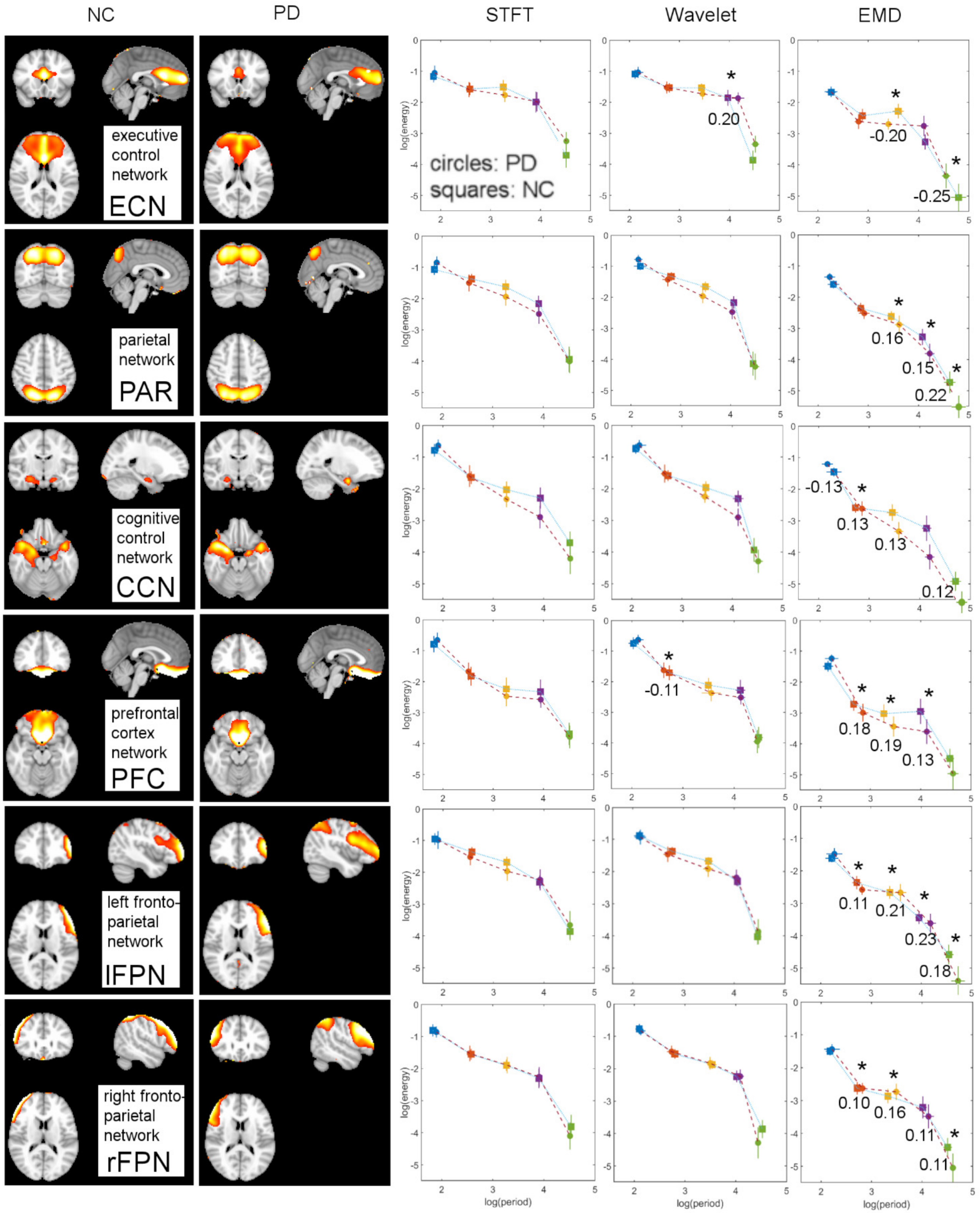

Different temporal characteristics of Fourier (STFT) components, wavelet (MODWT) components, and EMD components (IMFs) as measured by the $\log(energy)$ vs. $\log(period)$ relationship for six resting-state networks. The given numbers indicate the mean difference (over subjects) in $\log(period)$ between PD and NC. Only values in magnitude of at least 0.1 are shown (which was only possible in EMD analysis (20 instances) and MODWT (2 instances) but not in STFT (0 instances)). A "*" indicates a significant difference between PD and NC with $p<0.05$ (uncorrected). The horizontal and vertical bars indicate the standard deviation in $\log(period)$ and $\log(energy)$, respectively. Note that for most IMFs of the PAR, CCN, PFC, IFPN and rFPN, the mean period is larger for PD (circles) versus NC (squares), indicating that the decomposition of these networks by EMD leads to IMFs containing lower frequencies in PD than NC. Furthermore, most low frequency IMFs (i.e. IMFs with index >1) have lower energy for PD than NC.

References

Colominas, M., Schlotthauer, G., and Torres, M. E. 2014. Improved complete ensemble EMD: a suitable tool for biomedical signal processing. Biomed. Signal Process. Control 14, 19–29. doi: 10.1016/j.bspc.2014.06.009.

Dragomiretskiy, K. and Zosso, D. 2014. Variational Mode Decomposition. IEEE Transactions on Signal Processing, 62(3):531-544.

Fosso, O. B. 2019. Mode mixing separation in empirical mode decomposition of signals with spectral proximity. https://arxiv.org/pdf/1709.05547.pdf.

Huang, N.E. and Shen, S.S.P. 2014. Hilbert Huang Transform and its Applications. Interdisciplinary Mathematical Sciences, Vol.16. World Scientific Publishing Co. PTe. Ltd. Singapore.

Kaleem, M.F and Cordes, D. 2016. Demonstration of Effectiveness of a Novel-Data-Adaptive Method for fMRI Time Series Drift Removal. Organization of Human Brain Mapping Conference (HBM) Annual Meeting, Geneva, 2985.

Lin, S.-H. N., Lin, G.-H., Tsai, P.-J., Hsud, A.-L., Lo, M.-T., Yang, A.C., Lin, C.-P., Wu, C.W. 2016. Sensitivity enhancement of task-evoked fMRI using ensemble empirical mode decomposition. Journal of Neuroscience Methods 258: 56–66.

Moradi, N., Dousty, M., Sotero, R.C. 2019. Spatiotemporal Empirical Mode Decomposition of Resting-State fMRI Signals: Application to Global Signal Regression. Front. Neurosci., https://doi.org/10.3389/fnins.2019.00736.

Qian, L., Zhang, Y., Zheng, L., Shang, Y., Gao, J.-H., Liu, Y. 2015. Frequency Dependent Topological Patterns of Resting-state Brain Networks. PLOS ONE 10(4): e0124681.doi:10.1371/journal.pone.0124681.

Qian, L., Zheng, L., Shang, Y., Zhang, Y., Zhang, Y. 2018. Intrinsic frequency specific brain networks for identification of MCI individuals using resting-state fMRI. Neuroscience Letters, 664:7-14.

Riffi, J., Mahraz, A.M., Abbad, A., Tairi, H. 2015. 3D extension of the fast and adaptive bidimensional empirical mode decomposition. Multidimensional Systems and Signal Processing, 26:823–834.

Song, X., Zhang, Y., Liu, Y. 2014. Frequency Specificity of Regional Homogeneity in the Resting-State Human Brain. PLOS ONE 9(1):e86818.doi:10.1371/ journal.pone.0086818.

Torres, M. E., Colominas, M. A., Schlotthauer, G., and Flandrin, P. 2011. “A complete ensemble empirical mode decomposition with adaptive noise,” in Proceedings of the 36th IEEE International Conference on Acoustics, Speech and Signal Process, ICASSP 2011 (Prague), 4144–4147. doi: 10.1109/ICASSP.2011.5947265.

Yeh, J. R., Shieh, J. S., and Huang, N. E. 2010. Complementary ensemble empirical mode decomposition: a novel noise enhanced data analysis method. Adv. Adapt. Data Anal. 2, 135–156. doi: 10.1142/S1793536910000422.

Yuen, N.H., Osachoff, N., Chen, J.J. 2019. Intrinsic Frequencies of the Resting-State fMRI Signal: The Frequency Dependence of Functional Connectivity and the Effect of Mode Mixing. Front Neurosci.,13:900. doi: 10.3389/fnins.2019.00900.

Zhuang, X., Yang, Z., Mishra, V., Sreenivasan, K., Bernick, C., Cordes, D. (2020). Single-scale time-dependent window-sizes in sliding-window dynamic functional connectivity analysis: a validation study. Neuroimage 15;220:117111. doi: 10.1016/j.neuroimage.2020.117111.
